# Head and neck squamous cell carcinoma-derived extracellular vesicles mediate Ca²⁺-dependent platelet activation and aggregation through tissue factor

**DOI:** 10.1186/s12964-025-02215-x

**Published:** 2025-05-01

**Authors:** Tobias Weiser, Cosima C. Hoch, Julie Petry, Maria Shoykhet, Benedikt Schmidl, Mina Yazdi, Khouloud Hachani, Julia Mergner, Marie-Nicole Theodoraki, Omid Azimzadeh, Gabriele Multhoff, Ali Bashiri Dezfouli, Barbara Wollenberg

**Affiliations:** 1 Department of Otolaryngology, Head and Neck Surgery, TUM School of Medicine and Health, Technical University of Munich (TUM), Munich, Germany; 2 Pharmaceutical Biotechnology, Department of Pharmacy, Ludwig-Maximilians-Universität (LMU), Munich, Germany; 3 Bavarian Center for Biomolecular Mass Spectrometry at Klinikum rechts der Isar (BayBioMS@MRI), TUM School of Medicine and Health, Technical University of Munich, Munich, Germany; 4 Section Radiation Biology, Federal Office for Radiation Protection (BfS), Neuherberg, Germany; 5 Central Institute for Translational Cancer Research, Department of Radiation Oncology, TUM School of Medicine and Health, Technical University of Munich (TranslaTUM), Technical University of Munich, Munich, Germany

**Keywords:** Coagulation cascade, Cancer-associated thrombosis, Head and neck squamous cell carcinoma, Platelet activation, Extracellular vesicles, Tissue factor

## Abstract

**Background:**

Head and neck squamous cell carcinoma (HNSCC) is an aggressive malignancy, characterized by poor clinical outcomes, primarily driven by high rate of locoregional recurrence and metastasis. Extensive heterogeneity among the tumor cells as well as modulation of a highly immunosuppressive tumor microenvironment shape cancer progression. Shedding of extracellular vesicles (EVs) derived from tumor cells is a critical mediator of the disease initiating horizontal transfer of tumor components into platelets. This triggers platelet activation and thromboinflammation fueling tumor progression through multiple mechanisms.

**Methods:**

HNSCC-derived EVs isolated from HNSCC cell lines (SAS, UD-SCC 5) using size exclusion chromatography and characterized via flow cytometry, electron microscopy, nanoparticle tracking analysis and Western blotting, were used to induce platelet activation and aggregation, measured by aggregometry, flow cytometry, as well as the release of chemokines and Adenosine triphosphate, which were quantified using enzyme-linked immunosorbent assays (ELISA). Mechanistic investigations included inhibitor assays, thrombin activity measurements, and proteomic analyses.

**Results:**

We could show that EVs do not activate platelets through the FcγRIIa–IgG axis but platelet activation and aggregation is induced in a calcium-dependent manner, primarily mediated by EV-associated tissue factor. Proteomic analysis confirmed the presence of tissue factor in these vesicles, implicating its involvement in initiating the coagulation cascade, that leads to platelet activation and aggregation. This process was characterized by delayed aggregation kinetics and relied on thrombin activation, as the inhibition of thrombin and its receptors reduced platelet aggregation. HNSCC-derived EVs are pivotal in establishing a prothrombotic environment by promoting platelet activation and aggregation through tissue factor-dependent thrombin generation.

**Conclusion:**

These findings indicate a therapeutic potential of targeting EV-mediated pathways as a therapeutic approach to alleviate thrombotic complications in HNSCC patients. Subsequent animal studies will be crucial to validate and extend these observations, providing deeper insight into their clinical implications.

**Supplementary Information:**

The online version contains supplementary material available at 10.1186/s12964-025-02215-x.

## Introduction

Head and neck squamous cell carcinoma (HNSCC) represents an aggressive type of malignancy with limited therapeutic options in advanced stages [1]. High rates of recurrence and metastasis, along with resistance to therapy, contribute significantly to an unfavorable prognosis [2]. Continuous research efforts revealed an increasing body of evidence highlighting the crucial role of tumor interactions with the surrounding microenvironment in driving tumor progression, metastasis, and immune evasion [3, 4]. Among the various components of the tumor microenvironment (TME), extracellular vesicles (EVs), secreted by tumor cells, have emerged as important mediators of these interactions [5–8].

EVs are lipid bilayer-enclosed particles, that are released from cells. They are divided into small EVs (diameter < 200 nm) and large EV (diameter > 200 nm) [9, 10]. These vesicles serve as mediators of intercellular communication, facilitating the transfer of genetic material such as mRNAs and siRNAs, as well as bioactive molecules, including proteins, glycoconjugates, and lipids, between tumor cells and their surrounding environment [8, 11]. Recent studies, including the investigation of circulating EVs and their age-dependent influence on cell fate [12], emphasize the functional versatility of EVs in diverse physiological and pathological processes [13]. The two most extensively studied types of EVs are microparticles (MPs) and exosomes [14]. MPs are formed through shedding lipid raft regions of the plasma membrane in a calcium-dependent process involving the loss of membrane asymmetry and the exposure of phosphatidylserine [15, 16]. In contrast, exosomes are generated from multivesicular bodies in the cytosol that are released by exocytosis, interacting with recipient cells via protein interactions, membrane fusion, or endocytosis [17].

Tumor-derived EVs, particularly exosomes (TEX), are known for their role in modulating platelet (PLT) function, promoting PLT activation and aggregation and subsequently inducing the release of procoagulant factors [18–21]. While exosomes are known to participate in numerous tumor-associated processes, including angiogenesis, matrix remodeling, immunosuppression, and drug resistance, their specific involvement in cancer-associated thrombosis, especially in human studies, remains unclear [22]. Emerging evidence, however, suggests that TEX may contribute to a prothrombotic environment [20]. For example, elevated circulating TEX levels have been correlated with an increased risk of thrombosis in patients with advanced-stage ovarian cancer compared to those with early-stage disease or benign tumors [23].

Despite the growing knowledge of PLT supporting cancer progression, the precise mechanisms by which HNSCC-derived EVs induce PLT activation and aggregation remain poorly understood. This study aims to bridge this gap by investigating the functional properties of EVs from HNSCC cell lines as a standardized system. By characterizing the molecular cargo of these vesicles and their influence on PLT function, we aim to uncover new insights into the mechanisms by which EVs contribute to the prothrombotic environment in HNSCC, potentially in forming future therapeutic strategies.

## Materials and methods

### Reagents and chemicals

Tyrode’s buffer was prepared with the following components: 137 mM NaCl, 2 mM KCl, 12 mM NaHCO_3_, 0.3 mM NaH_2_PO_4_, 1 mM MgCl_2_, 5 mM HEPES, 5.5 mM glucose, and 0.5% bovine serum albumin (BSA), formulated with and without 2 mM CaCl_2_ (#793639, Merck). Phosphate-buffered saline (PBS)/Ethylenediaminetetraacetic acid (EDTA) was produced by adding 1 mM EDTA (#V4231, Promega) to PBS. The agonists Thrombin receptor activating peptide 6 (TRAP, #3497) and Innovin (#10284500) were purchased from Tocris Bioscience and Siemens Healthineers. The monoclonal antibody IV.3, generously provided by Ronald Taylor (University of Virginia), was used for specific blocking experiments. Prostaglandin I_2_ (PGI_2_, #P6188, Sigma) was employed as a PLT antagonist. Inhibitors used included U73122 (#J62898.MCR, ThermoFisher Scientific), Phe-Pro-Arg-Chloromethylketone (PPACK, #sc-201291, Santa Cruz Biotechnology), Hirudin (#H0393-100UN, Merck), Vorapaxar (#23119, Cayman Chemical), and BMS986120 (#23497-01, Cayman Chemical).

Fluorescence-conjugated monoclonal antibodies (mAbs) employed across various experiments including CD41 (#303710, #303729, BioLegend), CD62P (#304936, BioLegend), CD63 (#353030, BioLegend; #B92467, Beckman Coulter Life Sciences), CD142 (#365203, BioLegend), CD9 (#IM1755U, Beckman Coulter Life Sciences), and CD81 (#B25329, Beckman Coulter Life Sciences). For Western blot analyses, antibodies targeting CD9 (#10626D), Tumor susceptibility gene 101 (TSG101, #PA5-31260), and CD142 (#16-1429-82, also exhibiting blocking activity) were purchased from ThermoFisher Scientific. The antibodies against Glucose-regulated protein 94 (GRP94, #2104) and Glyceraldehyde 3-phosphate dehydrogenase (GAPDH, #5174) were obtained from Cell Signaling Technology. The complete list of antibodies, including their respective concentrations, is detailed in Table S1, Supporting Information.

### Cell lines and cell culture

HNSCC cell lines SAS and UD-SCC 5 (UD5), were cultured in Dulbecco’s Modified Eagle Medium (#21885108, ThermoFisher Scientific), supplemented with 10% fetal bovine serum (FBS, #10270106, ThermoFisher Scientific) and 100 U/mL Penicillin-Streptomycin (P/S, #P4333, ThermoFisher Scientific). Raji cells (Raji-Null, InvivoGen) were cultured in IMDM (#12440046, ThermoFisher Scientific) supplemented with 10% FBS and 100 U/mL P/S. For EV isolation, cells were washed twice with PBS and subsequently cultured in exosome depleted medium (EDM) for 24 to 48 h prior to EV collection. The EDM was prepared using Serum-Free Medium, (#20908-0500, CellGenix GmbH, Germany), supplemented with 10% Exosome-Depleted FBS (#A2720801, ThermoFisher Scientific), 100 U/mL Penicillin-Streptomycin, 1 mM sodium pyruvate (#11360070, ThermoFisher Scientific), and 2 mM L-Glutamine (#A2916801, ThermoFisher Scientific).

### Cell-derived EVs isolation from the culture medium

EV isolation was performed using Exo-spin™ midi columns (EX04, Cell Guidance Systems, St. Louis, USA), which employ size exclusion chromatography (SEC) to separate EVs, following the manufacturer’s protocol. Prior to application, the cell culture supernatant was concentrated using 50,000 Molecular weight cut-off (MWCO) Vivaspin filter tubes (#VS0641, Sartorius, Germany) by centrifugation at 2500 g at 4 °C. Then, 1 mL of concentrated cell culture supernatant was applied to the Exo-spin midi columns. After SEC, the isolated EVs were further concentrated using 100,000 MWCO filter tubes under the same centrifugation conditions. The concentration of the EVs was quantified using a BCA Protein Assay (#23227, ThermoFisher Scientific), performed following the manufacturer’s instructions. The isolated EVs were stored at 4 °C until use in the experiments.

### Characterization of EVs

Relevant data of our experiments have been submitted to the EV-TRACK knowledgebase (EV-TRACK ID: EV240184).

#### Nanoparticle tracking measurement

The particle size distribution of the isolated EVs was analyzed using nanoparticle tracking analysis (NTA) with the ZetaView PMX-130 instrument (Particle Metrix, Germany). Before measurement, EV samples were diluted in PBS to achieve a final particle concentration of 10^8^ particles/mL. Measurements were conducted at 25 °C using a 488 nm laser, with the camera sensitivity set to 80%. For each sample, 50 to 350 particles were counted per frame across 11 positions in each cycle. Data analysis was carried out using ZetaView software version 8.05.16.

#### Transmission electron microscopy imaging

A negative staining procedure was employed to visualize the structure of the isolated EVs. The EV samples were concentrated to a final concentration of 0.75 µg/µL using 100,000 MWCO ultrafiltration tubes (#VS0641, Sartorius). A 5 µL aliquot of the EV solution was placed onto a glow-discharged, carbon-coated copper transmission electron microscopy (TEM) grid and incubated for 1 min. The grids were then washed three times with droplets of distilled water to remove excess material. Following the washes, the grids were incubated three times with 3% uranyl acetate in water for 30 s per incubation. After air-drying, a thin layer of uranyl acetate residue remained on the grids. Imaging was performed using a JEOL 1400 transmission electron microscope (JEOL Ltd., Japan).

#### On bead flow cytometry

The surface expression of tetraspanins (CD9, CD63, CD81) and tissue factor (TF, CD142) on EVs was evaluated using flow cytometry. EVs were incubated overnight with capture beads (#ab239685, #ab239686, #ab239687, Abcam). After incubation, the EV-bead complexes were washed with PBS and stained with mAbs specific for CD9, CD63, CD81, and CD142 for 60 min at 4 °C under light-protected conditions. Following a final PBS wash, the stained EVs were analyzed using a flow cytometer, and fluorescence-labeled isotype-matched mAbs were used as controls.

To visualize and track EVs, they were pre-stained using the PKH67 Green Fluorescent Cell Linker Kit (#PKH67GL, Merck). Initially, EVs were isolated via SEC, recovered, and concentrated by ultracentrifugation at 150,000 g for at least 4 h at 4 °C. After discarding the supernatant, the EV pellet was resuspended in 1 mL of Diluent C, followed by the addition of 6 µL of PKH67 dye. The mixture was incubated for 5 min at RT, and the reaction was quenched by adding 2 mL of 10% BSA in PBS. The PKH67-stained EVs were then purified by layering them onto a sucrose cushion (1.5 mL of 0.971 M sucrose in PBS) and subjected to ultracentrifugation at 150,000 g for 2 h at 4 °C. After discarding the supernatant, the pellet was resuspended in 10 mL of PBS and further concentrated using 10,000 MWCO ultrafiltration tubes (#VS15T01, Sartorius) at 2500 g.

#### Western blotting of EVs

To evaluate the expression of TF (CD142) and EV specific markers (CD9 and TSG101), lysis buffer (10x; #9803, Cell Signaling Technology) was added to a final concentration of 1x to the isolated EVs. Following overnight incubation at -20 °C, the samples were thawed on ice, centrifuged at 10,000 g for 10 min at 4 °C, and supernatants were transferred to new tubes. The protein concentrations were determined using Bradford Assay (#5000001, Bio-Rad).

For application onto the sodium dodecyl sulfate (SDS) gel, the EV lysates were treated differently, according to the specificities of the antibodies. When analyzing TF, TSG101 or GRP94 together with the loading control GAPDH, Reducing Sample Buffer (#39000, ThermoFisher) was used, whereas for CD9 Non-Reducing Sample Buffer (#39001, ThermoFisher) was applied. All samples were boiled for 10 min at 95 °C. Subsequently, 20 µg of protein lysates were separated on a 12% SDS-PAGE gel and transferred onto nitrocellulose membranes (#1704270, ThermoFisher). Thereafter, the membranes were incubated overnight at 4 °C with the respective primary antibodies, and subsequently with species-matched horseradish peroxidase-conjugated secondary antibodies (#7074 and #7076, Cell Signaling Technology). Signals were developed using enhanced chemiluminescence substrate (#6883, Cell Signaling Technology) and visualized with the ChemiDoc XRS + system (Bio-Rad). Image analysis was performed using ImageLab 6.1 software (Bio-Rad).

### Platelet and plasma isolation

Venous blood was collected into tubes containing 3.2% sodium citrate (Sarstedt, Germany) to prevent clotting. For PLT isolation, PLT-rich plasma (PRP) was obtained by centrifugation at 150 g for 20 min. To obtain PLTs, prostaglandin I_2_ (PGI_2_, 400 nM) was added to the PRP, which was then diluted at a ratio of 2:1 with Tyrode’s buffer. A second centrifugation was performed at 800 g for 10 min. The supernatant was discarded, and the PLT pellet resuspended in 2 mL of Tyrode’s buffer. PLT count and purity were assessed using flow cytometry (CytoFLEX system, Beckman Coulter Inc., California, USA). Prior to experimentation, PLTs were allowed to rest at RT for at least 60 min. Washed PTLs were prepared by 3 additional centrifugation steps in PBS/EDTA at 450 g for 5 min each. To isolate plasma, the collected blood was first centrifuged at 1000 g for 10 min at RT. The resulting plasma was harvested and subjected to a second centrifugation at 2500 g for 10 min at RT. The supernatant was collected and used as plasma.

### Platelet viability assay

PLT viability and cell death were assessed using the Zombie Violet Fixable Viability Kit (#423114, BioLegend) following the manufacturer’s protocol. Briefly, washed PLTs were treated with EVs for 60 min at RT, with PBS as negative control and 80 µM Digitonin (#D5628, Sigma-Aldrich) as positive control. After treatment, the PLTs were washed twice with PBS/EDTA and stained with Zombie Violet (1:500 in PBS/EDTA) for 15 min at RT. Following a final PBS/EDTA wash, Zombie Violet positivity was detected by flow cytometry.

### EV uptake in platelets

#### Quantification of cellular uptake by flow cytometry

For quantification of EV uptake by PLTs, 200,000 PLTs/µL were co-incubated with 60 ng/µL of PKH67-stained EVs in a total volume of 100 µL of Tyrode’s buffer and incubated in the dark for 5 min, 2–4 h at RT. The PLTs were then fixed with 0.5% paraformaldehyde (PFA) in PBS for 15 min at RT, followed by washing with PBS/EDTA. After staining with a CD41-BV421 mAb for 15 min at RT, followed by an additional wash with PBS/EDTA, the PLT pellet was resuspended in Tyrode’s buffer and EV uptake was analyzed in flow cytometer.

#### Visualization of cellular internalization by fluorescent microscopy

Fresh PKH67-prestained EVs were used for immunofluorescent confocal imaging to evaluate the interaction between PLTs and EVs. PLTs were co-incubated with 60 µg/µL of either PKH67-stained or unstained EVs as a control in a total volume of 100 µL of Tyrode’s buffer, for 5 min, 2–4 h at RT in the dark. The PLTs were then fixed and stained as described in the section above. After an additional wash with PBS/EDTA, the PLT pellet was embedded in Fluorescent Mounting Medium (Agilent Technologies; #S3023) and mounted on slides. Images were acquired using a Leica TCS SP8 confocal laser-scanning microscope (Leica, Germany) with Leica LAS X software for analysis.

### Platelet functional assays

#### Aggregation assays

PLT aggregation was measured using the APACT 4 S PLUS aggregometer (DiaSys Diagnostic Systems GmbH). Aggregation results were quantified as the Area Under the Curve (AUC) or Maximal Aggregation (Max. Aggr.), with experiments conducted in a total volume of 200 µL and recorded for 1000 s. Following a 5-minute baseline recording, aggregation was initiated by the addition of specific agonists. In this study, the selected agonists were EVs (EVs, 60 µg/mL) and TRAP (25 µM). The aggregation lag time was defined as the duration from agonist addition to the point where a sustained increase in aggregation (lasting at least 5 s) was observed. For testing the aggregation involving plasma, 2 µL of homologous plasma (1%) was directly added to the preparations. Experiments with Innovin (final concentration: 100 ng/mL) were recorded over 1500 s following direct addition of the agonist to the platelet suspensions.

#### Inhibition studies

For inhibition studies, PLTs were pretreated with IV.3 antibody (300 ng/mL), U73122 (10 µM), Vorapaxar (10 µM), BMS986120 (10 µM), PPACK (1 µM), and Hirudin (2.5 U/mL) followed by the addition of the corresponding agonists. For the experiments involving the IV.3 antibody, heat-inactivated and immunoglobulin G (IgG)-coated *Escherichia coli* (ATCC^®^ 25922™; 5 × 10^7^/aggregometer run) served as control. They were prepared, with plasma incubated (IgG coating) and used as previously described [24].

#### Quantification of surface activation markers

PLT surface activation markers were analyzed following treatment with EVs at a concentration of 60 µg/mL in Tyrode’s buffer supplemented with 2 mM CaCl_2_. After the treatment, PLTs were fixed with 0.5% paraformaldehyde in PBS for 15 min at RT. The fixed PLTs were then washed with PBS/EDTA and incubated with CD41, CD62P, and CD63 antibodies at a dilution of 1:100 in PBS/EDTA for 15 min at RT, protected from light exposure. After incubation, the PLTs were washed, and the expression of surface markers was analyzed by flow cytometry.

#### Granule release, thrombin activity, and ATP quantification

To evaluate the release of the chemokine Chemokine ligand 5 (CCL5), Adenosine triphosphate (ATP), as well as thrombin activity, supernatants from PLTs treated with EVs were collected from the aggregometer at three different time points: 10 s after EVs addition, upon the onset of PLT aggregation and at 50% aggregation. Corresponding PBS controls were collected at the same time points. 2.5 µM PGI_2_ was added to the PLT suspensions to prevent further PLT degranulation. After centrifugation of the suspensions at 1000 g for 10 min at 4 °C, the supernatants were collected and stored at -80 °C till subsequent analysis.

For PLT-released CCL5 quantification, the supernatants were diluted 1:400 in Assay Diluent A and measured using a Legend Max Human CCL5 ELISA kit (#440804, BioLegend) according to the manufacturer’s instructions.

Thrombin activity was assessed using the Thrombin Activity Assay Kit (#ab234620, Abcam), following the manufacturer’s protocol. Briefly, 10 µL of each standard, PLT supernatant or plasma (treated with 60 µg/mL EVs) was combined with 90 µL of assay mix, and the reaction was incubated for 60 min at 37 °C. Then absorbance measurements were taken at 405 nm using a multimode plate reader (VICTOR Nivo, Revvity, Germany).

The ATP content in the supernatants was measured using the CellTiter-Glo Luminescent Cell Viability Assay (#G7571, Promega). The Supernatants were diluted 1:10 in Tyrode’s buffer, and 50 µL of the diluted sample or ATP standards were combined with 50 µL of CellTiter-Glo reagent in a 96-well plate. The mixture was shaken for 2 min to ensure thorough mixing and then incubated for 10 min at RT to allow luminescence development. Luminescence was recorded using a multimode plate reader.

### HNSCC cell characterization

#### Flow cytometric analysis of HNSCC cells

To assess TF (CD142) expression on the surface of adherent HNSCC cells (SAS and UD5 cell lines), the cells were washed and detached using TrypLE™ Express Enzyme (#12604-013, Thermo Fisher Scientific). 200,000 cells were resuspended in 100 µL PBS/EDTA and stained with CD142-PE antibodies for 15 min at RT, protected from light. After an additional wash, the cells were resuspended in PBS/EDTA and analyzed by flow cytometry.

#### Western blotting of HNSCC cells

Lysates from HNSCC cells were analyzed via Western blot to characterize the protein content. SAS and UD5 cells (10^6^ cells/well) were seeded in 6-well plates and cultured for 48 h. After cultivation, the cells were washed with PBS, and lysis buffer (final concentration 1x; #9803, Cell Signaling Technology) was added, followed by a 15-minute incubation on ice. The cell lysates were scraped from the plates and centrifuged at 10,000 g for 10 min at 4 °C. The supernatants were collected, and protein concentrations were measured using the Bradford Protein Assay (#5000001, Bio-Rad). Analysis of TF (CD142), TSG101, GAPDH, GRP94 and CD9 was performed as stated in section Western blotting of EVs.

### Tracking of the tissue factor by imaging flow cytometry

For imaging flow cytometry, to visualize TF, fixed PLTs were labeled with CD41-BV421 to identify PLTs, while CD142-PE was used to label the TF present on the EVs. The staining was performed for 15 min at RT. After washing with PBS/EDTA, images were acquired using the ImageStream Mk II system (MilliporeSigma, Germany) at 60× magnification. The data were analyzed using AMNIS IDEAS 6.2 Software. In this experiment, brightfield images were acquired in Channel 1, TF (CD142-PE) in Channel 3, and PLTs (CD41-BV421) in Channel 7. The number of CD142-PE spots resulting from the interaction between PLTs and EVs was quantified using the Spot Count feature.

### Proteomic analysis of EVs

To analyze the protein content of SAS EVs, samples were sent for proteomic analysis. Sodium deoxycholate was added to a final concentration of 2% to isolated EVs in PBS from 5 biological replicates and boiled for 10 min at 95 °C. 20 µg protein input was reduced (Dithiothreitol, 10 mM), alkylated (chemical alkylating agent, 55 mM) and digested with trypsin [2 × 1:100 (wt/wt) enzyme-to-protein ratio] at 37 °C overnight. Digested samples were acidified with formic acid and desalted on self-made C18 StageTips [25]. Liquid chromatography-coupled mass spectrometry (LC-MS/MS) analysis was performed on an Eclipse mass spectrometer (Thermo Fisher Scientific) coupled on-line to a Dionex Ultimate 3000 RSLCnano system using a 50 min linear gradient and data independent acquisition mode. DIA-NN [26] version 1.8.1 was used to generate an in-silico predicted spectral library composed of the human proteome (UniprotKB reference proteome, UP000005640, download 01/2021) and common contaminants (MaxQuant contaminants.fasta) with trypsin as digestion enzyme and one missed cleavage specified. Subsequently the acquired raw files were processed in library-free mode using DIA-NN default settings and the match between runs function enabled. The R package iq [27] was used to calculate MaxLFQ protein intensity values. If not stated otherwise, all protein abundance values refer to log2 transformed MaxLFQ protein intensity values. The proteomic profiling was performed using Enrichr [28] and STRING database [29].

### Statistical analysis

Data analysis was performed using GraphPad Prism (version 10.1.1). To test for normal distribution of the data, the Shapiro-Wilk test was applied. Statistical significance of normally distributed data was assessed using unpaired Student’s *t*-tests (two groups) and One-way ANOVAs or Mixed Models (more than two groups), followed by post-hoc tests such as Tukey’s, Bonferroni’s, or Dunnett’s, as appropriate. Non-parametric distributed data were analyzed using the Mann-Whitney test (two groups) and the Kruskal-Wallis test (more than two groups), followed by Dunn’s post-hoc test. Two-way ANOVA was applied when comparing more than two groups with two variables, followed by Tukey’s post-hoc tests. The results are expressed as mean + standard deviation. The p-values were considered statistically significant as ns – not significant, *p ≤ 0.05, **p ≤ 0.01, ***p ≤ 0.001, and ****p ≤ 0.0001.

## Results

### Physiochemical characterization of HNSCC-derived EVs

**Fig. 1:**
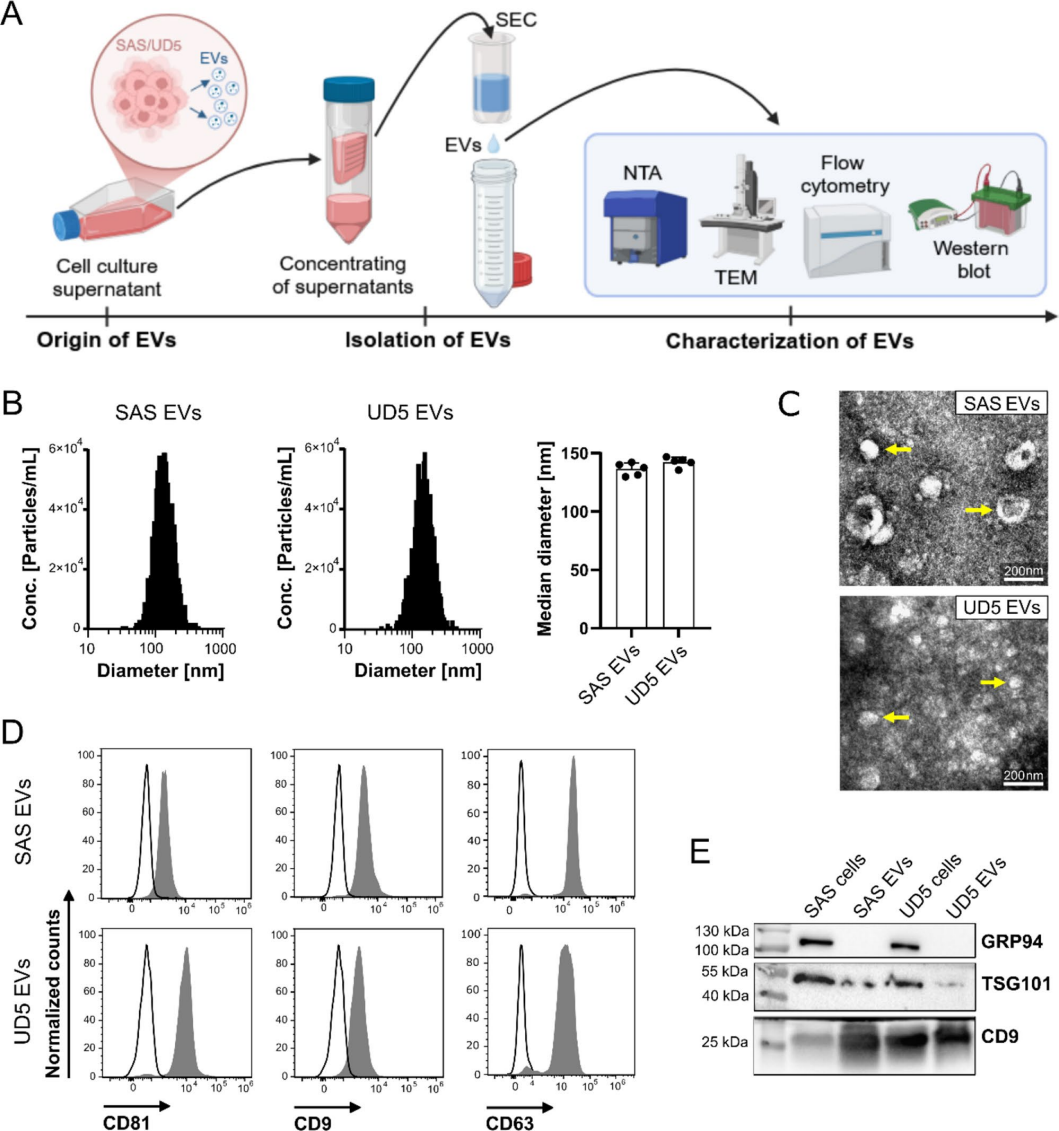
Characterization of EVs isolated from HNSCC cell lines. EVs were isolated from the cell culture supernatant of SAS and UD5 cells using Exo-spin™-based size exclusion chromatography (SEC). **(A)** Schematic overview of the EV isolation and characterization process. This graph was created using BioRender.com. **(B)** Size distribution profiles of the isolated EVs as determined by NTA. The bar chart illustrates the median size of SAS and UD5 EVs (*n* = 5 independent NTA analyses). **(C)** Representative TEM images displaying the morphological characteristics of the EVs. The scale bars (white) are 200 nm. **(D)** Representative flow cytometric histograms of typical EV surface markers (gray-shaded histograms) using fluorescein isothiocyanate (FITC)-conjugated tetraspanins (CD9, CD63, CD81) monoclonal antibodies. Their respective isotype-matched mAbs were used as negative controls (black line/empty histograms). **(E)** Representative Western blot images of EV (CD9 and TSG101) and cellular (GRP94) markers from SAS and UD5 whole cell and respective cell culture-derived EV protein lysates. 20 µg of lysates were loaded per lane. The molecular weight markers (in kDa) provide size references, indicating the molecular weights of CD9 (~ 25 kDa), TSG101 (~ 44 kDa) and GRP94 (~ 100 kDa)

Tumor-derived EVs were isolated from HNSCC cell lines SAS and UD5 cultured in EDM, utilizing Exo-spin™ midi columns for SEC. The EVs were characterized in accordance with the MISEV criteria [10] and evaluated using protein content analysis, NTA, and TEM, as outlined in the schematic representation in Fig. 1A. NTA analysis revealed that the diameter of EVs from both cell lines ranged between 50 and 300 nm, with a mean particle size of 136.2 ± 5.4 nm for SAS-derived EVs and 142.3 ± 4.2 nm for UD5-derived EVs (Fig. 1B). TEM images confirmed the morphology, integrity, and approximate size range of the EVs, validating their three-dimensional spherical structure with size range of 100–200 nm, which is consistent with the NTA measurements (Fig. 1C). Protein composition and vesicular biomarkers were analyzed by flow cytometry and Western blot. Flow cytometric analysis confirmed the presence of EV-typical tetraspanins CD9, CD63, and CD81 on the surface of EVs derived from SAS and UD5 cells (Fig. 1D). Western blot analysis showed the presence of CD9 and the exosomal marker TSG101 and the absence of GRP94 in EVs. The non-exosomal endoplasmic reticulum protein GRP94, was exclusively detected in whole-cell lysates from both cell lines (Fig. 1E).

### Ca²⁺-dependent platelet activation and aggregation induced by EVs

**Fig. 2:**
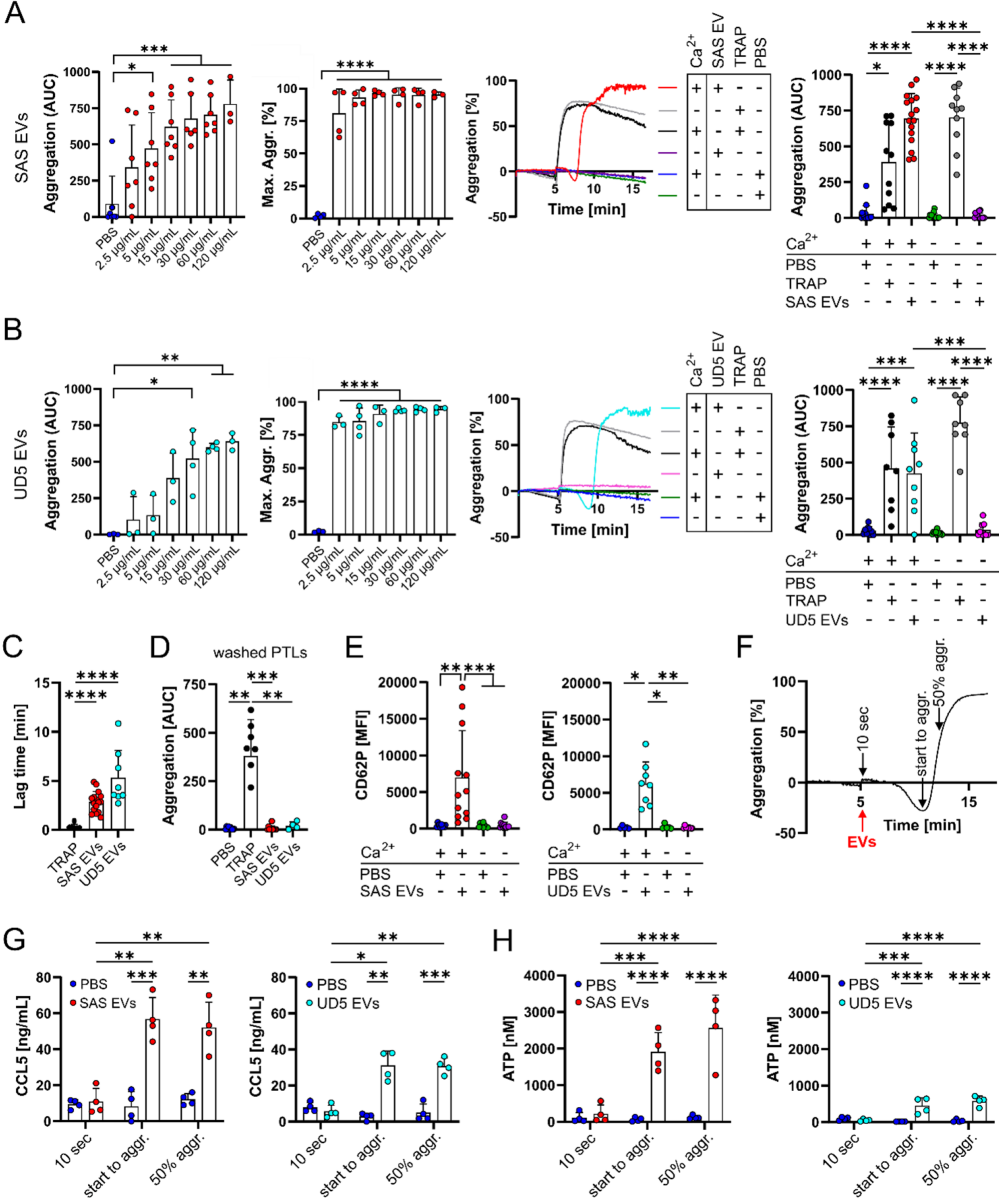
Ca^2+^-dependent induction of PLT aggregation and activation by HNSCC-derived EVs. **(A)** PLT aggregometry following exposure to SAS-derived EVs at varying concentrations. PLT aggregation was measured using an aggregometer. After a 300-second baseline stirring period, EVs were added at 2.5, 5, 15, 30, 60, and 120 µg/mL concentrations. PLT aggregation was continuously monitored for 1000 s. PBS, the elution buffer used during SEC for EV isolation, was a negative control. The first graph quantifies aggregation as the area under the curve (AUC) for each condition (*n* = 3–7). The second bar graph illustrates the percentage of maximal PLT aggregation observed within 1000 s across varying concentrations of SAS-derived EVs (*n* = 3–4). The third graph shows representative light transmission aggregation curves for PLTs exposed to 60 µg/mL SAS EVs, TRAP (positive control (ctrl) to induce aggregation) and PBS in Tyrode’s buffer, either with 2 mM Ca²⁺ (+) or without Ca²⁺ (**-**). The fourth graph presents an AUC-based quantitative PLT aggregation analysis, after treatment with SAS EVs (60 µg/mL) or PBS and TRAP as controls (*n* = 9–11). **(B)** PLT aggregometry following exposure to UD5-derived EVs at varying concentrations. PLT aggregation was measured using an aggregometer. After a 300-second baseline stirring period, EVs were added at 2.5, 5, 15, 30, 60, and 120 µg/mL concentrations. PLT aggregation was continuously monitored for 1000 s. PBS, the elution buffer used during SEC for EV isolation, was a negative control. The first graph quantifies aggregation as the area under the curve (AUC) for each condition (*n* = 3–4). The second bar graph illustrates the percentage of maximal PLT aggregation observed within 1000 s across varying concentrations of UD5-derived EVs (*n* = 3–4). The third graph shows representative light transmission aggregation curves for PLTs exposed to 60 µg/mL UD5 EVs, TRAP (positive ctrl to induce aggregation) and PBS in Tyrode’s buffer, either with 2 mM Ca²⁺ (+) or without Ca²⁺ (-). The fourth graph presents an AUC-based quantitative PLT aggregation analysis, after treatment with UD5 EVs (60 µg/mL) or PBS and TRAP as controls (*n* = 8–10). **(C)** Lag time analysis of PLT aggregation induced by SAS- and UD5-derived EVs (*n* = 8–13) compared to TRAP (positive ctrl). **(D)** Measurement of aggregation potential in triple-washed PLTs after addition of SAS and UD5 EVs (60 µg/mL), PBS or TRAP (*n* = 4–11). **(E)** Flow cytometric analysis of PLT activation using CD62P as a marker. The graph shows the mean fluorescence intensity (MFI) values for CD62P expression, indicating PLT activation. PLTs were incubated with SAS- or UD5-derived EVs, or PBS (negative control), in Tyrode’s buffer, with or without 2 mM Ca²⁺ (*n* = 10–12, left graph; *n* = 5–8, right graph). **(F)** Schematic of time points for supernatant collection during PLT-EV coincubation: 10 s after EV addition (10 s), at the onset of aggregation (start to aggr.), and 50% of maximum aggregation (50% aggr.). **(G)** CCL5 release, measured by ELISA from PLT supernatants collected at specific time points, indicates PLT degranulation following incubation with 60 µg/mL of SAS- or UD5-derived EVs (*n* = 4). **(H)** ATP assay conducted on PLT supernatants following incubation with SAS- or UD5-derived EVs (60 µg/mL, *n* = 4). Data are represented as mean **+** Standard deviation (SD), with statistical significance generally denoted as follows: *p ≤ 0.05, **p ≤ 0.01, ***p ≤ 0.001, ****p ≤ 0.0001. One-way ANOVA followed by Tukey’s post-hoc test for (A, B) and by Bonferroni’s post-hoc test for (B, third graph). Kruskal-Wallis test followed by Dunn’s post-hoc test for (A, third graph) and (C-E). Mixed Model followed followed by Dunett’s post-hoc test for (B, second and third graph). Two-way ANOVA followed by Tukey’s post-hoc test for (G, H)

The effect of HNSCC-derived EVs on aggregation, was tested incubating varying concentrations of EVs with PLTs. To mimic physiological conditions, PLTs were resuspended in Tyrode’s buffer containing 2 mM Ca²⁺, reflecting the measured average Ca²⁺ concentration in donor serum and plasma (Figure S1A, Supporting Information), before analysis in an aggregometer. EVs induced PLT aggregation in a concentration-dependent manner (Fig. 2A, B, first graphs; Figure S1B, Supporting Information), whereas the maximum aggregation level was already reached at an EV-concentration of 2.5–5 µg/mL across all tested EVs, indicating that higher EV concentrations enhanced the aggregation induction without increasing the maximum capacity. All EVs yielded a significantly greater aggregation compared to the phosphate buffered saline (PBS) control, with over 85% aggregation achieved at a concentration of 60 µg/mL across the majority of samples analyzed (Fig. 2A, B, second graphs). Based on these findings, 60 µg/mL of EVs was selected for subsequent experiments to ensure robust induction of PLT aggregation. To further investigate the dependency of EV-induced PLT aggregation on Ca^2+^, experiments were conducted with the established EV dose in Tyrode’s buffer both with and without Ca^2+^ supplementation. Results demonstrated that SAS and UD5 cell line-derived EVs induced aggregation exclusively in the presence of Ca^2+^, whereas TRAP, used as a positive control, induced PLT aggregation (AUC and maximum level of aggregation) regardless of the availability of Ca^2+^ (Fig. 2A, B, third and fourth graphs; Figure S1C,D, Supporting Information). In contrast, Tyrode’s buffer containing 2 mM Ca^2+^ in the absence of EVs failed to trigger PLT aggregation. A detailed analysis of the aggregation kinetics revealed that the SAS and UD5 EV-induced PLT aggregation exhibited a delayed onset compared to TRAP (Fig. 2C). The average aggregation lag time was 2.85 min for SAS-derived EVs and 5.31 min for UD5-derived EVs, representing delays of more than 8-fold and 15-fold, respectively, compared to the average lag time of 0.33 min for TRAP (Fig. 2C). This pronounced delay suggests that EVs may not directly activate PLTs. Notably, additional washing steps of PTLs abolished the EV-induced aggregation, unlike TRAP-induced aggregation (Fig. 2D), indicating that the aggregation effect might involve residual coagulation factors. To further confirm the essential role of remaining plasma coagulation factors, triple-washed PLTs were incubated with or without a defined amount of orthologous plasma (1%), and aggregation was detected in the presence and absence of Ca^2+^ in the Tyrode’s buffer. Unlike the positive control TRAP, SAS and UD5- derived EVs could only induce aggregation in the presence of plasma and Ca^2+^ (Figure S1E, Supporting Information). The lack of either one of the two components prevents EV-induced aggregation. An additional confirmation that aggregation-relevant plasma coagulation factors are present on the regular PLT preparation and removed by washing was obtained by adding Innovin, which is a lipidated recombinant tissue factor that is used for assessing coagulant activity. Innovin induce aggregation only in the standard PLT preparation, whereas triple-washed PLTs remained unresponsive (Figure S1F, Supporting Information).

The observed aggregation delay and wash-sensitive nature of EV-induced aggregation support the hypothesis that the coagulation cascade may play a role in the EV-induced PLT activation. Additionally, PLT activation following exposure to EVs in Tyrode’s buffer containing Ca^2+^ was confirmed via flow cytometry by assessing the surface expression of CD62P and CD63. Both activation markers show a significant increase upon coincubation of PLTs with SAS- and UD5-derived EVs (Fig. 2E; Figure S1G, H, Supporting Information). To further confirm PLT activation, granule release was measured via CCL5 ELISA (measure of alpha granule release) and ATP levels (dense granule release) with PLT supernatants following respective treatments with SAS- and UD5-derived EVs. These supernatants were acquired at three time points post-EV treatment (Fig. 2F). Both CCL5 (Fig. 2G) and ATP levels (Fig. 2H) demonstrated a notable increase after coculturing of PTLs with SAS and UD5 EVs. These findings confirmed the hypothesis that PLTs release α-granules and dense granules when coincubated with EVs in the presence of Ca^2+^. Taken together, the results show that SAS- and UD5-derived EVs effectively induce PLT aggregation, activation, and granule release in a Ca^2+^-dependent manner.

### EV toxicity and time-based progression of uptake in PLTs

**Fig. 3:**
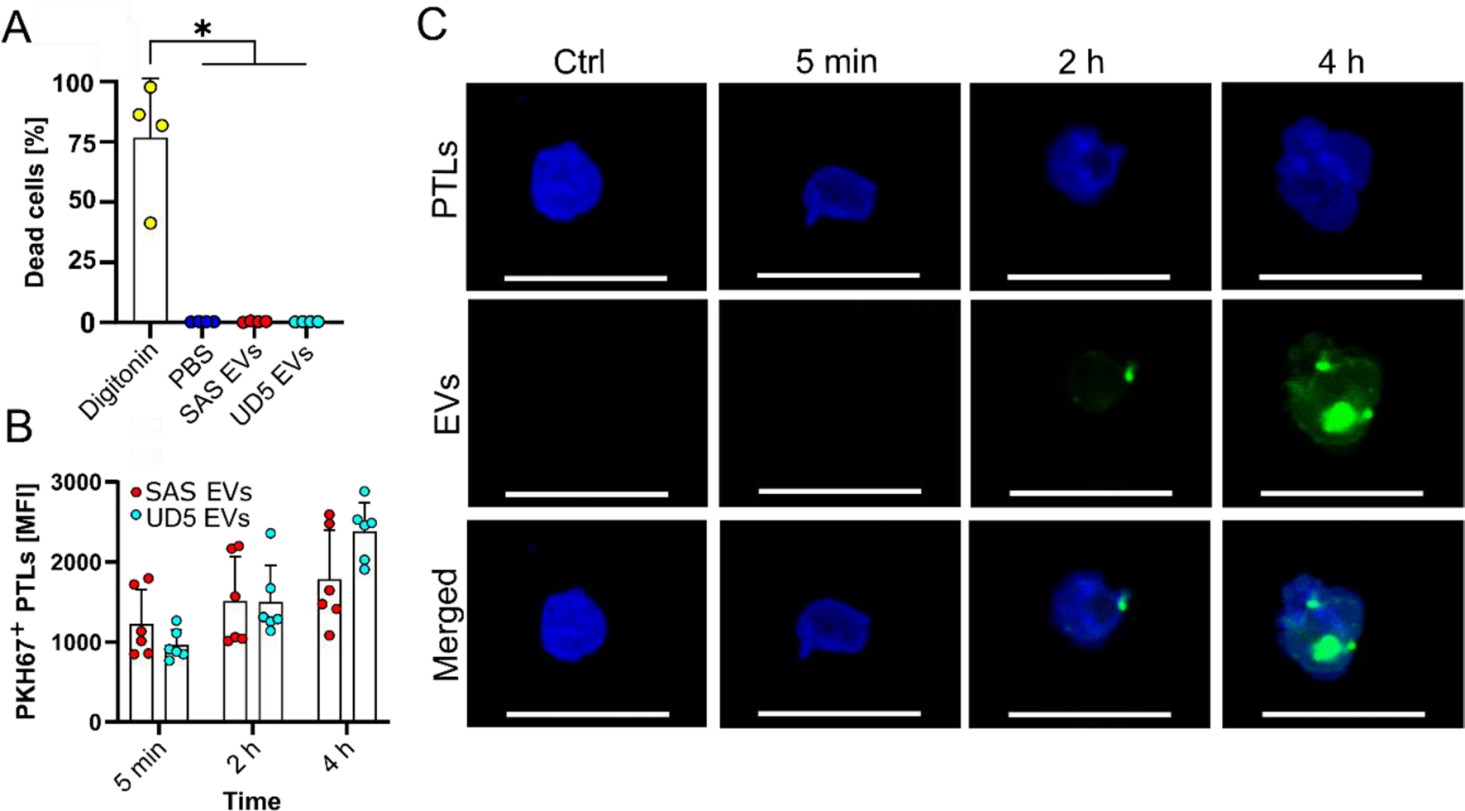
Toxicity evaluation and time-dependent uptake of EVs by PLTs. **(A)** Assessment of PLT viability using Zombie Violet live-dead staining. PLTs were incubated with 60 µg/mL EVs in Tyrode’s buffer without Ca^2+^. Digitonin (80 µM) and PBS-treated groups were used as controls. PLTs treated with EVs and lacking Zombie violet staining were used to establish gating parameters. The graph represents the percentage of Zombie violet-positive (dead) cells. Data are presented as mean + SD. Statistical significance is indicated as follows: *p < 0.05 (*n* = 4), determined using a One-way ANOVA followed by Tukey’s post-hoc test. **(B)** Quantification of PLT-EV interactions was performed using flow cytometry. PLTs were incubated with 60 µg/mL of PKH67-labeled SAS- and UD5-derived EVs in Tyrode’s buffer without Ca^2+^ supplementation, and the PKH67 fluorescence intensity was measured in 5 min, 2 h and 4 h post-co-culture. Following staining with CD41-BV421 antibody, PKH67-positive PLTs were quantified. **(C)** Representative confocal microscopy images of PLTs incubated with PKH67-labeled (green) SAS-derived EVs (60 µg/mL) for 5 min, 2 h and 4 h. PLTs were stained with CD41-BV421 (blue) to visualize PLT morphology. Unlabeled EVs incubated for 4 h served as a control. The scale bar (white) represents 10 μm

The potential toxicity of the selected effective dose of EVs (60 µg/mL) on PLTs was assessed via a cell viability assay using Zombie Violet staining, following a 60-minute coincubation period. Across all conditions, a low toxicity was observed compared to digitonin (80 µM), a detergent used as a positive control for cell death (Fig. 3A). This indicates that the tested EV dose did not induce PLT toxicity. The interaction between EVs and PLTs was further analyzed using flow cytometry and confocal microscopy. PLTs were coincubated with PKH67-labeled EVs, and PKH67 positivity was measured to quantify EV uptake over time. Minimal or no PKH67 positivity was detected 5 min after treatment. However, an increase in PKH mean fluorescence intensity (MFI) was observed after 2 and 4 h, suggesting a gradual interaction between EVs and PLTs over time (Fig. 3B). Additionally, confocal microscopy was used to visualize the interaction of PKH-labeled EVs and PLTs. Representative images of internalized EVs are shown after 5 min, 2 h, and 4 h of coincubation (Fig. 3C).

### Mechanisms of EV-induced platelet aggregation and downstream signaling cascade

**Fig. 4:**
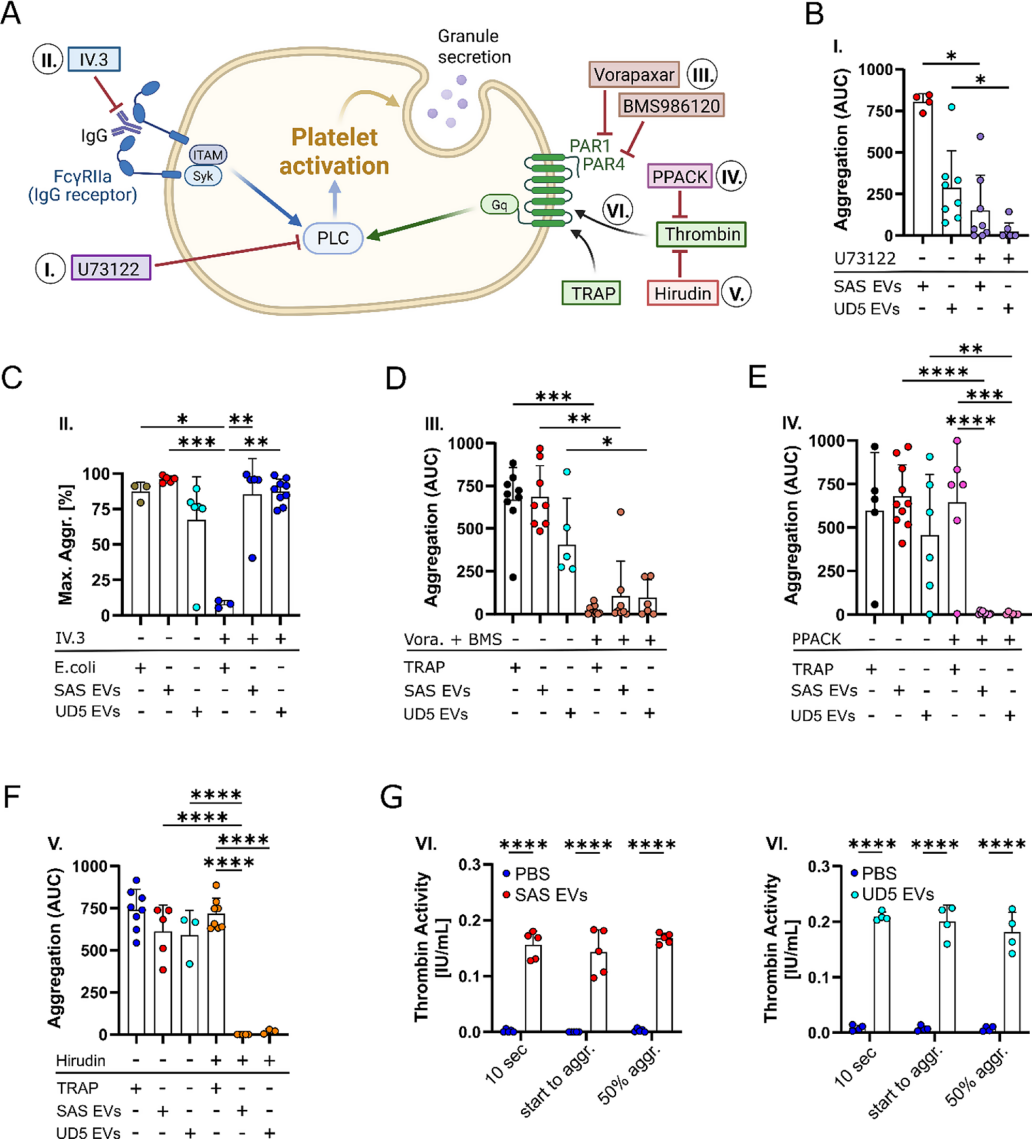
EV-induced PLT aggregation and activation involve the coagulation cascade. PLTs were suspended in Tyrode’s buffer containing 2 mM Ca^2+^, and aggregation was monitored for 1000 s. **(A)** Schematic overview of used PLT aggregation inhibitors and their targets within the PLT activation and aggregation pathways. This graph was created using BioRender.com. **(B)** Role of phospholipase C (PLC) in EV-induced PLT aggregation. PLTs were pre-incubated with U73122 (10 µM; +; PLC inhibitor), without U73122 (-) or Dimethyl sulfoxide (DMSO; 1:250 dilution; ctrl) for 300 s, followed by the addition of SAS- or UD5 derived EVs (60 µg/mL). PLT aggregation was quantified as the area under the curve (AUC, *n* = 4–8). **(C)** Effect of FcγRIIa inhibition on PLT aggregation. PLTs were pre-incubated with (+) or without (-) the FcγRIIa-blocking antibody IV.3 (300 ng/mL) for 300 s in an aggregometer. IgG-coated *E. coli* (5 × 10⁷ bacteria/sample) or SAS and UD5 EVs (60 µg/mL) were then added to assess FcγRIIa-dependent PLT activation (*n* = 3–9). **(D)** Involvement of PAR1 and PAR4 thrombin receptors in EV-induced PLT aggregation. PLTs were pre-treated with Vorapaxar (+; Vora.; 10 µM; PAR1 inhibitor) and BMS986120 (+; BMS; 10 µM; PAR4 inhibitor) or vehicle control (-; DMSO, 1:250 dilution) for 300 s, followed by the addition of SAS or UD5-derived EVs (60 µg/mL). Thrombin receptor activation was induced using TRAP (25 µM; thrombin receptor agonist) as a positive control (*n* = 5–11). **(E)** PLTs were treated with the inhibitor PPACK (+, 1 µM) or DMSO control (-, 1:20,000 dilution) to block thrombin activity, and aggregation was assessed following stimulation with SAS and UD5 EVs (60 µg/mL) or TRAP (25 µM; positive control) (*n* = 5–10). **(F)** PLT aggregation following treatment with the thrombin inhibitor hirudin (+, 2.5 µM) or H_2_O control (-, 1:200 dilution) was **measured** after stimulation with SAS and UD5 EVs (60 µg/mL) or TRAP (25 µM) (*n* = 3–8). **(G)** Thrombin activity during PLT aggregation. Thrombin activity (IU/mL) was measured at 10 s, the start of aggregation, and at 50% aggregation. PLTs were incubated with SAS- or UD5-derived EVs (60 µg/mL), and supernatants were collected for analysis. PBS-treated PLTs served as controls (*n* = 5, left graph; *n* = 4, right graph). Data are presented as the mean + SD from *n* ≥ 3 independent experiments. Statistical significance: *p < 0.05, **p < 0.01, ***p < 0.001, ****p < 0.0001. Kruskal-Wallis test followed by Dunn’s post-hoc test for (B-D). One-way ANOVA followed by Bonferroni’s post-hoc test for (E, F). Two-way ANOVA followed by Tukey’s post-hoc test for (G)

The mechanism underlying EV-induced PLT aggregation was tested by employing a series of inhibitors targeting key components of the coagulation cascade and PLT activation pathways, in the presence of Ca²⁺ in Tyrode’s buffer, as outlined in the scheme shown in Fig. 4A. First, we examined the involvement of Phospholipase C (PLC) in EV-mediated aggregation. The PLC inhibitor U73122 (10 µM, establishment of U73122 concentration, Figure S2A, Supporting Information) led to a significant reduction in EV-induced PLT aggregation (Fig. 4B), indicating that PLC functions as a downstream component in the EV-mediated PLT aggregation pathway. Since FcγRIIa is a major platelet receptor for IgG-containing complexes and signals upstream of PLC, we next assessed its role in EV-induced aggregation. To test whether the EVs trigger aggregation via IgG, we blocked this pathway using the monoclonal antibody IV.3. Unlike IgG-coated E. coli cell-induced aggregation (control), EV-triggered aggregation was unaffected by FcγRIIa inhibition (Fig. 4C), suggesting that EVs induce PLT aggregation independently of IgG delivery and the FcγRIIa receptor. Building on the established role of PLC, we further investigated the involvement of upstream thrombin receptors, specifically protease-activated receptors 1 (PAR1) and PAR4. To investigate this, we applied the PAR1 inhibitor Vorapaxar and the PAR4 inhibitor BMS986120 (each 10 µM) in parallel to check for potential synergistic effects. Pre-incubation of PLTs with the single inhibitors Vorapaxar or BMS986120 alone, did not hinder EV-induced PLT aggregation (Figure S2B, Supporting Information). When both thrombin receptor inhibitors were combined a marked reduction in SAS- and UD5-derived EV-induced PLT aggregation was observed (Fig. 4D; Figure S2B, Supporting Information). Furthermore, inhibition of thrombin itself with PPACK (1 µM, establishment of inhibitory PPACK concentration: Figure S2C, Supporting Information) or Hirudin (2.5 µM, determination of inhibitory Hirudin concentration: Figure S2D, Supporting Information) suppressed PLT aggregation induced by both SAS- and UD5-derived EVs (Fig. 4E, F), underscoring thrombin’s critical role in the aggregation process. To validate the involvement of thrombin, we measured thrombin activity upon incubation of PLTs with SAS- and UD5-derived EVs. This was assessed in PLT supernatants collected at three time points post-EV treatment (Fig. 2F). A significant increase in thrombin activity was observed within 10 s after the addition of EVs (Fig. 4G), reinforcing thrombin’s key role in EV-induced PLT aggregation. The addition of SAS- and UD5-derived EVs to human plasma also induced thrombin activity, although PBS treatment as a control did not differ significantly (Figure S2E, Supporting Information). Collectively, these findings indicate that thrombin activation is essential for EV-induced PLT aggregation, with downstream components, such as the PLT thrombin receptors PAR1/PAR4 and Phospholipase C, also being crucial in this process.

### EVs mediate platelet aggregation initialized by tissue factor (CD142)

The increased thrombin activity after the addition of EVs to PLTs, along with the delayed start of aggregation compared to TRAP, suggest that the coagulation cascade may play a role in EV-induced PLT aggregation. To explore the involvement of the coagulation pathway in EV-induced aggregation initiation, we conducted a representative proteomic analysis of SAS-derived EVs. The analysis identified 2189 proteins, among those, 1227 proteins were identified with at least 2 unique peptides and detected in 4 out of 5 replicates (Table S2, Supporting Information). Proteomic profiling of SAS EVs indicated several biological pathways related to the structure and function of PLTs (Figure S3; Table S3, Supporting Information). The cluster of proteins belonging to coagulation was the second enriched pathway (p-value: 1.54E-38 and q-value: 3.85E-37; Figure S3A; Table S3, Supporting Information) with 62 proteins involved in regulation of coagulation, complement activation and PLTs alpha-granule release (Figure S3B and Table S3, Supporting Information). TF (CD142) was also identified in SAS-derived EVs and was particularly associated with the positive regulation of coagulation.

**Fig. 5:**
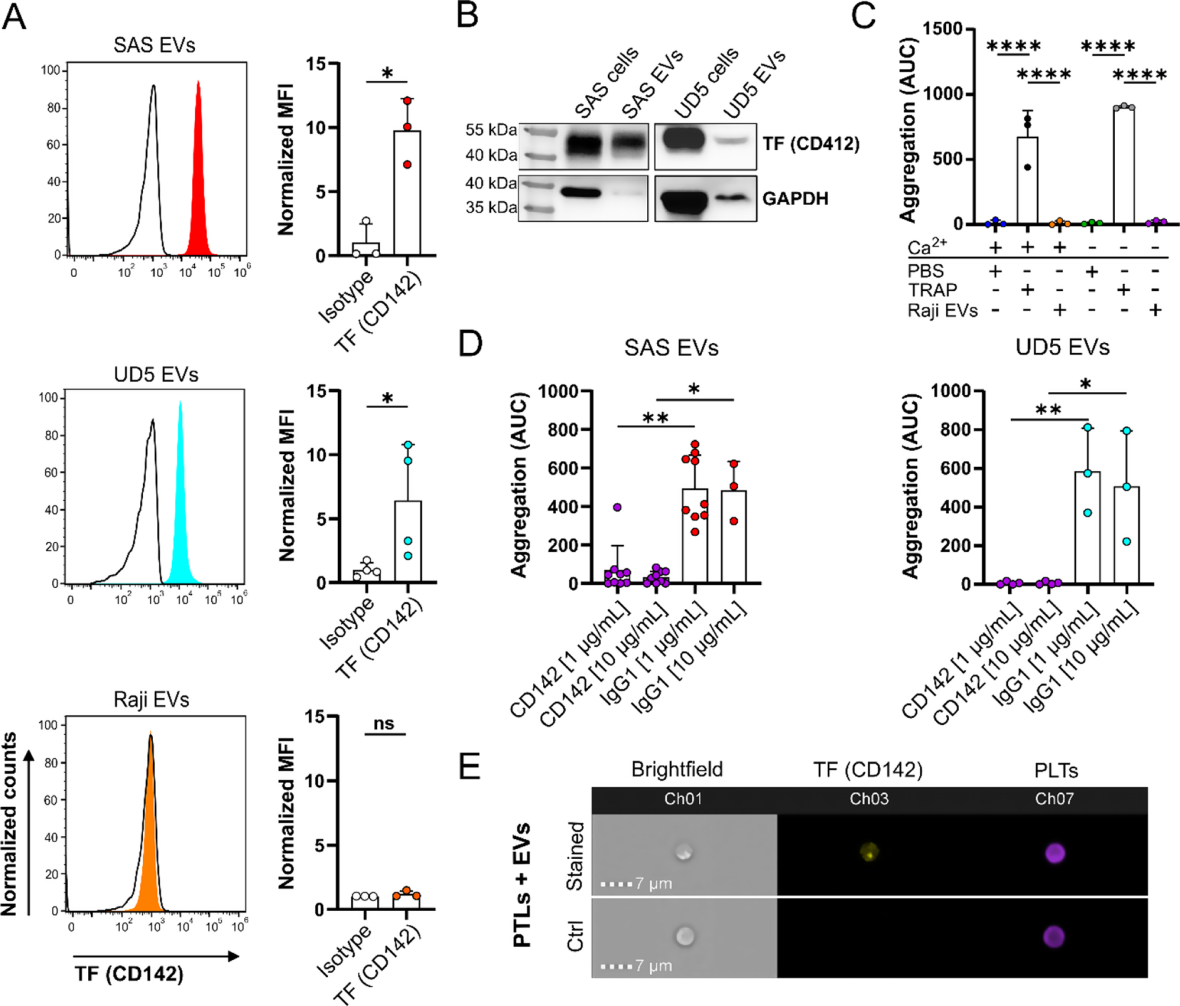
HNSCC EVs promote platelet aggregation through activation of the coagulation cascade mediated by Tissue Factor (CD142). **(A)** Flow cytometric analysis of TF (CD142) expression on EVs isolated from SAS (*n* = 3), UD5 (*n* = 4) and Raji cells (*n* = 3). The left panels display representative histograms of CD142-PE staining (colored peaks) compared to the matched isotype controls (black lines) from SAS EVs, UD5 EVs and Raji EVs. The data are normalized to mode for comparison. The right panels summarize the MFI of TF expression normalized to the isotype control for each EV-type. **(B)** Western blot analysis of TF (CD142) protein expression in SAS cells, SAS EVs, UD5 cells and UD5 EVs. The molecular weight markers (in kDa) indicate bands corresponding to TF (~ 47 kDa) and GAPDH (~ 36 kDa, loading control). **(C)** The effect of Raji-derived EVs on PLT aggregation was evaluated using aggregometry. PLTs were suspended in Tyrode’s buffer containing 2 mM Ca²⁺ (+) or without Ca²⁺ (-), and aggregation was monitored following the addition of Raji-derived EVs (60 µg/mL) at 300 s. TRAP was used as positive control to induce PLT aggregation, while PBS was considered as negative control (*n* = 3). **(D)** Inhibition of SAS- and UD5-derived EV-induced PLT aggregation following mAb-mediated blockade of TF (CD142). SAS (*n* = 3–9) and UD5 EVs (*n* = 3–4) were first pre-incubated with CD142 mAb (1 µg/mL and 10 µg/mL), and IgG1 as a matched isotype was used as a negative control. The pre-incubated EVs (60 µg/mL) were subsequently added to PLTs in the aggregometer at the 300 s time point. **(E)** Representative images of TF (CD142) internalization analyzed by Imaging flow cytometry. PLTs were incubated with SAS-derived EVs (60 µg/mL) for 5 min and analyzed across three channels: bright field (Ch01), TF-associated SAS-derived EVs labeled with CD142-PE (yellow, Ch03), and PLTs labeled with CD41-BV421 (purple, Ch07). The interaction between PLTs and EVs was compared to an unstained control. The scale bar (dashed line) is 7 μm. Data are presented as mean + SD. Statistical significance is as follows: ns-*p* > 0.05, *p < 0.05, **p < 0.01, ****p < 0.0001. Mann-Whitney test for (A, first and third graph) and unpaired Student’s *t*-test for (A, second graph). One-way ANOVA followed by Tukey’s post-hoc test for (C) and (D, second graph). Kruskal-Wallis test followed by Dunn’s post-hoc test for (D, first graph)

The presence of TF (CD142) on the surface of SAS- and UD5-derived EV and cell samples was analyzed via flow cytometry and Western blot. The gating strategy of the relevant cell populations is illustrated in Figure S4A (Supporting Information). Flow cytometric analysis demonstrated that both SAS and UD5 cells and their respective EVs express TF (CD142) on their surface (Fig. 5A; Figure S4B, Supporting information). Western blot analysis of SAS and UD5 cells, as well as their EVs, further confirmed the presence of TF (CD142) in the examined samples (Fig. 5B). Raji cells are a human B-lymphocyte cell line with tumorigenic properties [30] and their EVs were utilized as negative controls for TF (CD142) expression. The quality of Raji cell-derived EVs was assessed through NTA analysis (Figure S4C, Supporting Information), flow cytometry (Fig. 5A, S4D, Supporting Information), and Western blot (S4E, Supporting Information). These analyses revealed a typical average median size of Raji EVs and the presence of the tetraspanins CD63 and CD81 in flow cytometry analysis and CD9 in the Western blot analysis. In contrast to HNSCC-derived EVs, Raji cells and their EVs were tested as negative for TF in flow cytometry a, d Western blots (Fig. 5A; S4E, Supporting Information). Notably, Raji-derived EVs did not induce PLT aggregation when co-incubated with PTLs in Tyrode’s buffer containing 2 mM Ca^2+^ (Fig. 5C). Based on these findings, we performed a blocking study to validate the role of TF in EV-induced PLT aggregation. Blocking of TF (CD142) with a specific antibody significantly inhibited SAS and UD5 EVs aggregation. Both antibody concentrations tested (1 µg/mL and 10 µg/mL) significantly prevented PLT aggregation compared to IgG1-treated controls (Fig. 5D; S4F, Supporting Information). Having confirmed the key role of TF (CD142) in PTL activation, CD41-labeled PLTs cocultured with TF-labeled EVs were used to track the uptake of TF (CD142). After 5 min of coincubation, PLTs were fixed, stained, and analyzed. ImageStream cytometry (Imaging flow cytometry) quantified TF uptake or binding by capturing images of individual PLTs and detecting TF-positive spots. Upon EV contact, punctate fluorescent spots appeared, indicating EV-associated TF uptake or binding by PLTs, with each spot representing a distinct event. An average of 0.63 ± 1.11 TF-positive spots per cell were observed, while unstained EV-treated control PLTs showed no positivity (Fig. 5E). These findings confirm that TF signals can be detected on PLTs within the time frame relevant for EV-driven aggregation and activation.

## Discussion

**Fig. 6:**
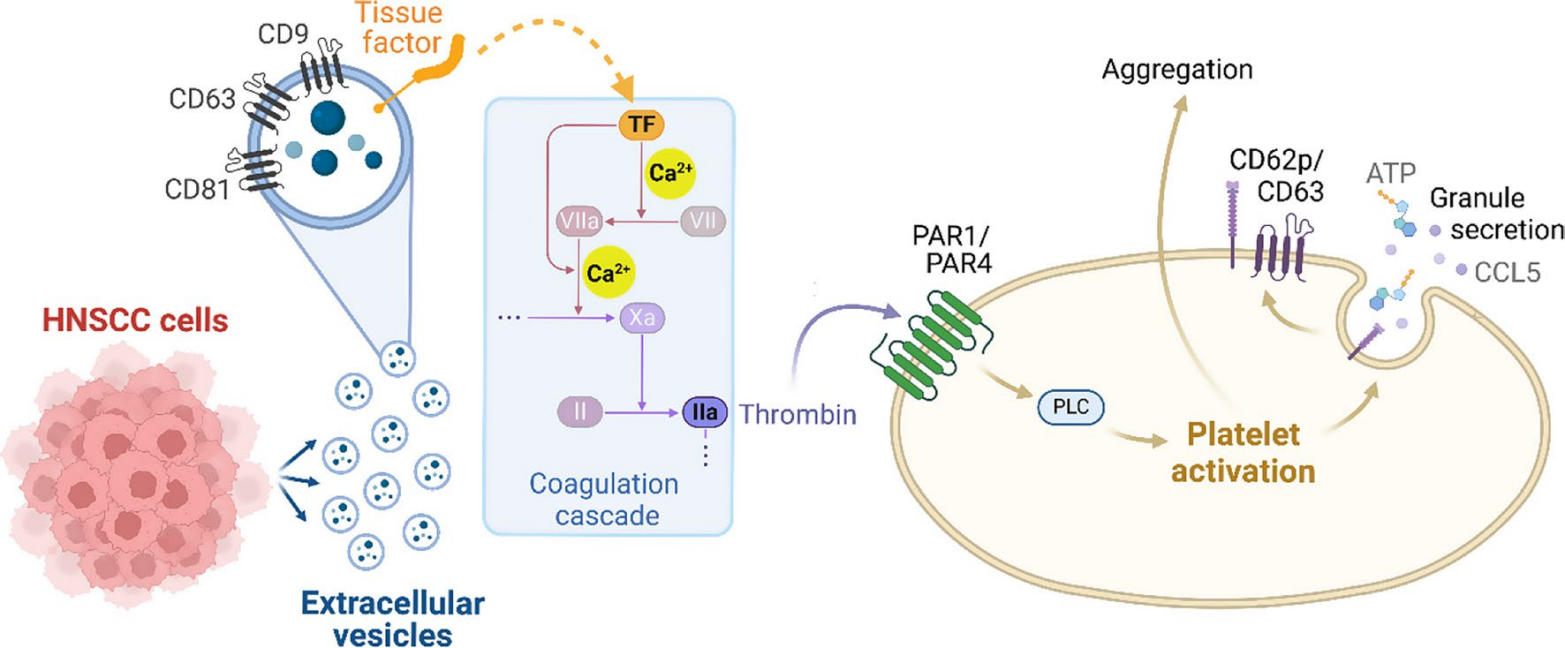
Graphical summary of the investigated mechanism - EVs released by HNSCC cells promote PLT activation and contribute to a pro-thrombotic environment. HNSCC cells produce EVs enriched with surface markers such as CD9, CD63, and CD81, as well as TF (CD142). Once released, these EVs interact with plasma components, leading to the production thrombin (factor IIa), potentially via the coagulation cascade. Thrombin then binds to protease-activated receptors (PAR1 and PAR4) on the PLT surface. This interaction initiates intracellular signaling pathways, such as those mediated by phospholipase C, which drive PLT granule secretion. As a result, activated PLTs release pro-inflammatory mediators, such as ATP and CCL5 and express activation markers such as CD62P and CD63 on their surface, further amplifying PLT aggregation and reinforcing the thrombotic environment. This graphical summary was created using BioRender.com

HNSCC is an aggressive malignancy characterized by high rates of metastasis and recurrence, with the TME playing a crucial role in these processes [3, 4]. EVs have emerged as important mediators of intercellular communication within the TME, facilitating the transfer of bioactive molecules that influence immune responses, enhance tumor invasiveness, and contribute to the hypercoagulable state observed in cancer patients. Our findings reveal that HNSCC-derived EVs present TF on their surface and thereby initiate the coagulation cascade. This process leads to thrombin generation and subsequent Ca²⁺-dependent PTL activation, aggregation and granule release, as illustrated in the graphical abstract (Fig. 6).

Ensuring the identity and quality of EVs is essential for understanding their biological effects. The isolation method plays a crucial role in determining the purity and integrity of EVs. We utilized the Exo-spin SEC kit, a gentle, size-exclusion based separation technique that yields highly pure, homogenous vesicles with minimal protein contamination [31, 32]. In contrast, EVs isolated via ultracentrifugation, though widely used are more heterogenous and often contain co-isolation contaminants, such as protein aggregates, lipoproteins (e.g., HDL, LDL), and other MPs, which can result in unspecific aggregation [33]. The quality of Exo-spin isolated EVs was confirmed by the presence of exosomal markers such as tetraspanins (CD9, CD63, CD81) and TSG101. Morphologically, the EVs exhibited a round, circular shape with a diameter size between 135 nm and 245 nm, which is consistent with established EVs criteria [9]. The concentrations of EVs applied in studies vary depending on the experimental objectives. For evaluations focusing on functional impact, concentrations typically ranging from 0.1 to 10 µg/mL have been used, with optimal results frequently observed at approximately 10 µg/mL [34, 35]. Other studies have employed higher EV concentrations, ranging from 12.5 to 100 µg/mL, particularly for assays investigating cellular uptake, signal transduction, immunomodulatory effects, and other biological responses [36–38]. In our experiments, EVs were tested in a concentration range of 2.5 to 120 µg/mL. Low concentrations of 2.5 and 5 µg/mL EVs were sufficient to trigger PLT aggregation; to ensure a robust PLT aggregation for all experiments a non-lethal concentration of 60 µg/mL was used.

PLTs can be activated via several receptors on their surface. Among these, the PLT FcγRIIa mediates activation by binding immune complexes containing IgG [39, 40]. Our findings suggest that HNSCC-derived EVs do not activate PLTs through the FcγRIIa–IgG axis. This conclusion is supported by the observation that blocking FcγRIIa with the monoclonal antibody IV.3 did not reduce EV-induced PLT aggregation. This implies that the procoagulant activity of EVs is independent of immune complex-dependent mechanisms, commonly observed in EVs from other malignancies [41–44]. A plausible explanation is that HNSCC-derived EVs may lack significant IgG content, due to mechanisms governing vesicle biogenesis. Alternatively, the surface composition of these vesicles may inherently preclude activation through the FcγRIIa pathway.

Our study demonstrates that HNSCC cell line-derived EVs likely activate PLTs through TF-mediated pathways. TF, a key initiator of the extrinsic coagulation cascade, binds to factor VIIa, to form a complex that activates downstream coagulation factors, including thrombin. Thrombin subsequently binds to PAR1 and PAR4 receptors on PLTs, triggering their activation and promoting aggregation [45]. We demonstrated that HNSCC cells are TF-positive, as are EVs, which were isolated using SEC. Blocking TF (CD142) with an anti-TF (CD142) antibody significantly reduced PLT aggregation and activation, whereas TF-negative EVs from Raji cells failed to induce aggregation. Our observations build upon previous research linking TF-positive EVs to cancer-associated thrombosis and cancer progression [46, 47]. For instance, elevated levels of TF-positive MPs have been associated with an increased risk of venous thromboembolism in multiple malignancies [36, 37, 48–51]. Moreover, analyses of TF-positive EV activity in human plasma samples have shown a strong correlation between elevated TF levels and an increased risk of thrombosis [52]. In the context of HNSCC, Adesanya et al. reported that tumor-derived MPs reduce clotting time and upregulate TF expression in human umbilical vein endothelial cells (HUVECs) [53]. Their study demonstrated an indirect procoagulant effect mediated through HUVECs, whereas our investigation offers direct and compelling evidence of TF expression on well-characterized EVs.

Our experiments revealed that co-incubation of HNSCC cell line-derived EVs with PLTs induces robust PLT aggregation and activation as evidenced by notable increase in aggregation as measured by aggregometry, enhanced secretion of alpha and dense granules, which is quantified by elevated levels of ATP and CCL5 in ELISA, and a marked increase in the expression of surface activation markers, specifically CD62P and CD63. The observed effect was dependent on the presence of extracellular Ca²⁺. It is essential to note that Ca²⁺ alone, when evaluated as a control, did not induce PLT aggregation or activation during our experiments. Moreover, extensive washing of PLTs to remove residual coagulation factors abolished the EV-induced aggregation and activation. The addition of orthologous plasma could restore this loss of function. These observations suggest that Ca²⁺ is essential for EV-induced PTL activation and aggregation within the observed time period. Ca²⁺ could act in concert with EV-associated TF and other coagulation elements. This aligns with established mechanisms of PLT activation, wherein calcium ions are pivotal in the coagulation cascade [54–56]. Additionally, PLT activation itself depends on elevated cytosolic Ca²⁺ levels, which increase through either the release of intracellular stores or the influx of Ca²⁺ across the plasma membrane [57, 58]. Consistent with our findings, studies on tumor cell-induced PLT aggregation have revealed that various tumor cells, such as those from hepatocellular and gastric carcinomas, can elevate intracellular Ca²⁺ levels in PLTs and induce aggregation through thrombin generation and the involvement of coagulation factors [59].

The delayed onset of aggregation observed in our experiments suggests that EVs do not act as direct PLT agonists but rather initiate a series of downstream events leading to thrombin activation. This temporal delay distinguishes EV-mediated PLT activation from that induced by classic agonists, such as TRAP, which rapidly and directly activate PLTs. While EV-induced PLT aggregation was delayed compared to TRAP, it ultimately resulted in stronger overall aggregation. Granule release was similarly delayed, occurring a few minutes after EV addition. Our findings indicate that HNSCC cell line-derived EVs initiate the coagulation cascade before PLT activation. Specifically, we observed a rapid increase in thrombin activity within seconds of EV exposure, suggesting that EVs rapidly generate thrombin, which then drives PLT activation. This aligns with previous research demonstrating the importance of thrombin in EV-induced and prostate cancer-related PLT aggregation [37, 38]. Moreover, the inhibition of thrombin using PPACK and Hirudin or blocking both thrombin receptors reduced EV-induced PLT aggregation.

Our investigation into the interaction between HNSCC cell line-derived EVs and PLTs revealed minimal direct binding or uptake of EVs by PLTs during the initial stages of aggregation. This finding suggests that the procoagulant activity of EVs is likely mediated by soluble factors, such as thrombin, rather than through direct vesicle-PLT interactions. These observations align with previous studies that underscore the importance of coagulation factors in cancer-associated PLT activation [60, 61]. MPs are known to exhibit greater procoagulant activity than activated PLTs, primarily due to their enhanced capacity to bind coagulation factors [62]. We propose that TF on EVs serves as the primary driver of the observed procoagulant activity, though additional mechanisms may be involved. One such mechanism could be the exposure of phosphatidylserine on the EV surface, which is known to accelerate coagulation processes. This hypothesis is supported by the rapid generation of thrombin detected in our study, coupled with the lack of significant EV uptake by PLTs within the first 5 min of exposure. Collectively, these observations suggest a model in which HNSCC cell line-derived EVs promote thrombin generation, subsequently engaging PAR1/PAR4 receptors on PLTs, thereby initiate their activation and aggregation.

Despite the significant findings of this study, several limitations should be acknowledged. First, our experimental setup relied on in vitro models to investigate the effects of HNSCC cell line-derived EVs on PLT activation and coagulation. While these models provide valuable mechanistic insights, they may not fully capture the complexity of the TME in vivo and the dynamic interactions occurring within the circulatory system of cancer patients. Future studies should incorporate in vivo models to validate our findings and assess the clinical relevance of EV-mediated PLT activation in a more physiologically relevant context. Second, the EVs concentration used in our experiments was chosen to ensure robust PLT aggregation and activation. Although these concentrations fall within the range reported in the literature [63, 64], they may not accurately reflect physiological EV concentrations in the plasma of HNSCC patients [65]. Lastly, our study did not examine EVs derived from patient-derived HNSCC cells or primary tumors. Instead, we employed established HNSCC cell lines, which may limit the generalizability of our findings, as EVs from primary tumors could exhibit different molecular and functional profiles. Future research should consider using patient-derived EVs to better understand the variability and clinical implications of EV-mediated PLT activation in HNSCC patients.

## Conclusion

This study demonstrates that HNSCC cell line-derived EVs play a pivotal role in promoting a prothrombotic environment by inducing PLT activation and aggregation in a Ca²⁺-dependent manner. These EVs present TF on the surface, which initiates the extrinsic coagulation cascade, drives thrombin generation, and subsequently promotes PLT aggregation. This activation occurs via a delayed mechanism distinct from traditional agonists, highlighting the unique role of EV-associated TF in orchestrating coagulation processes. The minimal direct interaction between EVs and PLTs and the abolition of aggregation upon washing further underscore the involvement of soluble coagulation factors rather than direct vesicle-PLT binding. The findings provide insights into the procoagulant mechanisms associated with HNSCC and present promising opportunities for the targeted modulation of EV-dependent pathways to mitigate thrombotic complications and tumor progression in cancer patients [46]. In vivo studies utilizing patient-derived EVs have the potential to validate these outcomes and could elucidate their capacity to enhance clinical results.

## Electronic supplementary material

Below is the link to the electronic supplementary material.

Supplementary Material 1

Supplementary Material 2

Supplementary Material 3

Supplementary Material 4

## Data Availability

All data generated or analyzed during this study are included in the published article and its supplementary information or will be available from the corresponding author upon reasonable request. The mass spectrometry proteomics data have been deposited to the ProteomeXchange Consortium via the PRIDE [66] partner repository with the dataset identifier PXD060122.

## Abbreviations

ATP: Adenosine triphosphate
AUC: Area under the curve
BSA: Bovine serum albumin
CCL5: Chemokine ligand 5
Ctrl: Control
DIA: Data-independent acquisition
DMSO: Dimethyl sulfoxide
EDM: Exosome depleted medium
EDTA: Ethylenediaminetetraacetic acid
EV: Extracellular vesicles
FBS: Fetal bovine serum
FITC: Fluorescein isothiocyanate
GAPDH: Glyceraldehyde 3-phosphate dehydrogenase
GRP94: Glucose-regulated protein 94
HNSCC: Head and neck squamous cell carcinoma
IgG: Immunoglobulin G
LC: MS/MS-Liquid chromatography-tandem mass spectrometry
mAb: Monoclonal antibody
MP: Microparticle
MWCO: Molecular weight cut-off
NTA: Nanoparticle tracking analysis
PAR: Protease-activated receptors
PBS: Phosphate buffered saline
PGI_2_: Prostaglandin I_2_
PLC: Phospholipase C
PPACK: Phe-Pro-Arg-chloromethylketone
PLT: Platelets
RT: Room temperature
SD: Standard deviation
SEC: Size exclusion chromatography
SDC: Sodium deoxycholate
SDS: Sodium dodecyl sulfate
TEM: Transmission electron microscopy
TEX: Tumor-derived exosomes
TF: Tissue factor (CD142, F3)
TME: Tumor microenvironment
TRAP: TRAP-6 (Thrombin receptor activating peptide 6)
TSG101: Tumor susceptibility gene 101
UD5: UD-SCC 5 (University of Dusseldorf-Squamous Cell Carcinoma-5)
U: Units
WB: Western blot
