# Supplementary material for: Head and neck squamous cell carcinoma-derived extracellular vesicles mediate Ca²⁺-dependent platelet activation and aggregation through tissue factor
Source: Cell Commun Signal. 2025 May 1;23:210. doi: 10.1186/s12964-025-02215-x (PMC12044835; doi:10.1186/s12964-025-02215-x)
Supplement: Supplementary file 1 — Supplementary Material 1 [file 12964_2025_2215_MOESM1_ESM.docx]

**Supplementary Tables & Figures**

**Table S1. List of antibodies used in this study.** The table provides information about the target antigen, the concentration applied in experiments, the specific application the antibodies were used for and the respective suppliers.

| **Target** | **Concentration** | **Application** | **Ref** | **Supplier** |
| --- | --- | --- | --- | --- |
| **Anti-mouse** | 20 ng/mL | WB | #7076 | Cell Signaling Technology |
| **Anti-rabbit** | 15 ng/mL | WB | #7074 | Cell Signaling Technology |
| **CD142** | 1 - 10 µg/mL | Aggregometry | #16-1429-82 | ThermoFisher Scientific |
|  | 1 µg/mL | WB |  |  |
| **CD142** | 1 µg/mL | Flow cytometry | #365203 | Biolegend |
| **CD41** | 500 ng/mL | Flow cytometry | #303710 | Biolegend |
| **CD41** | 1 µg/mL | IF | #303729 | Biolegend |
| **CD62p** | 1 µg/mL | Flow cytometry | #304936 | Biolegend |
| **CD63** | 250 ng/mL | Flow cytometry | #353030 | Biolegend |
| **CD63** | 10 µg/mL | Flow cytometry | #B92467 | Beckman Coulter Life Sciences |
| **CD63** | 500 ng/mL | WB | #10628D | ThermoFisher Scientific |
| **CD81** | 10 µg/mL | Flow cytometry | #B25329 | Beckman Coulter Life Sciences |
| **CD9** | 500 ng/mL | WB | #10626D | ThermoFisher Scientific |
| **CD9** | 10 µg/mL | Flow cytometry | #IM1755U | Beckman Coulter Life Sciences |
| **GAPDH** | 35 ng/mL | WB | #5174 | Cell Signaling Technology |
| **GRP94** | 1:1000 dilution | WB | #2104 | Cell Signaling Technology |
| **IV.3** | 300 ng/mL | Aggregometry |  | provided by Ronald Taylor |
| **TSG101** | 3 µg/mL | WB | #MA5-32463 | ThermoFisher Scientific |

**Table S2.** **All proteins identified in SAS EV proteomics.** The proteins in bold were identified with at least two unique peptides and detected in four out of five replicates (Supplementary Material 2).

**Table S3.** **The top enriched pathways ranked by -log10(p-value) and the related proteins. (**Supplementary Material 3).

**
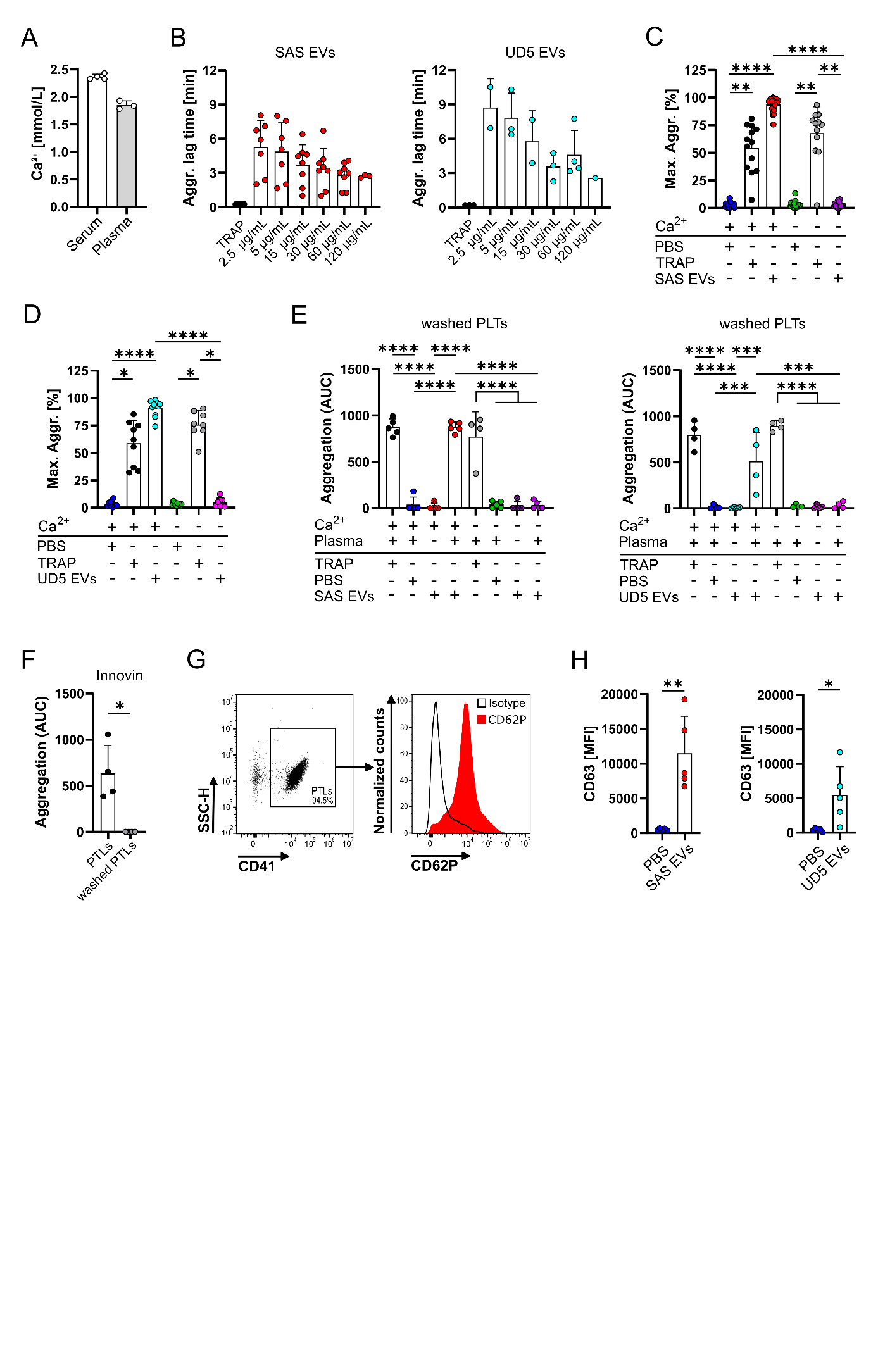
**

**Figure S1. Ca²⁺-dependent platelet activation and aggregation induced by HNSCC-derived EVs.** **(A)** Measurement of Ca²⁺ levels in the serum (n = 4) and plasma (n = 3) of blood donors used for PLT isolation. **(B)** PLT aggregation lag time was measured in the presence of 2 mM Ca²⁺ after treatment with varying concentrations of SAS- and UD5-derived EVs. **(C, D)** Maximal PLT aggregation induced by HNSCC cell line-derived EVs. Aggregation following treatment with SAS- and UD5-derived EVs is shown. PLTs in Tyrode’s buffer, with (+) or without (−) 2 mM Ca²⁺, were treated with 60 µg/mL EVs. TRAP and PBS served as controls (n = 12-21, C; n = 8-11, D). **(E)** The aggregation of triple-washed platelets (PBS/EDTA, washed PLTs) was measured in the presence (+) or absence (−) of 1% autologous plasma after the addition of SAS and UD5 EVs (60 µg/mL). Platelets were resuspended in Tyrode’s buffer either containing (+) or lacking (−) 2 mM Ca²⁺. TRAP and PBS were used as controls. **(F)** To test Innovin-induced (100 ng/mL) aggregation, experiments were conducted in Tyrode’s buffer without Ca²⁺ using PLTs and triple-washed platelets (PBS/EDTA, washed PLTs). **(G)** Representative gating strategy for identifying CD62P expression on CD41-positive PLTs. **(H)** Analysis of CD63, a PLT activation marker. CD63 expression is displayed after treatment of PLTs with SAS- and UD5-derived EVs (60 µg/mL, n = 5). Data are presented as mean + SD. Statistical significance is as follows: *p < 0.05, **p < 0.01, ***p < 0.001, ****p < 0.0001. Kruskal-Wallis test followed by Dunn’s post-hoc test for (C, D). One-way ANOVA followed by Tukey’s post-hoc test for (E). Mann-Whitney test for (F) and (H, SAS EVs) and unpaired Student’s *t*-test for (H, UD5 EVs).

**
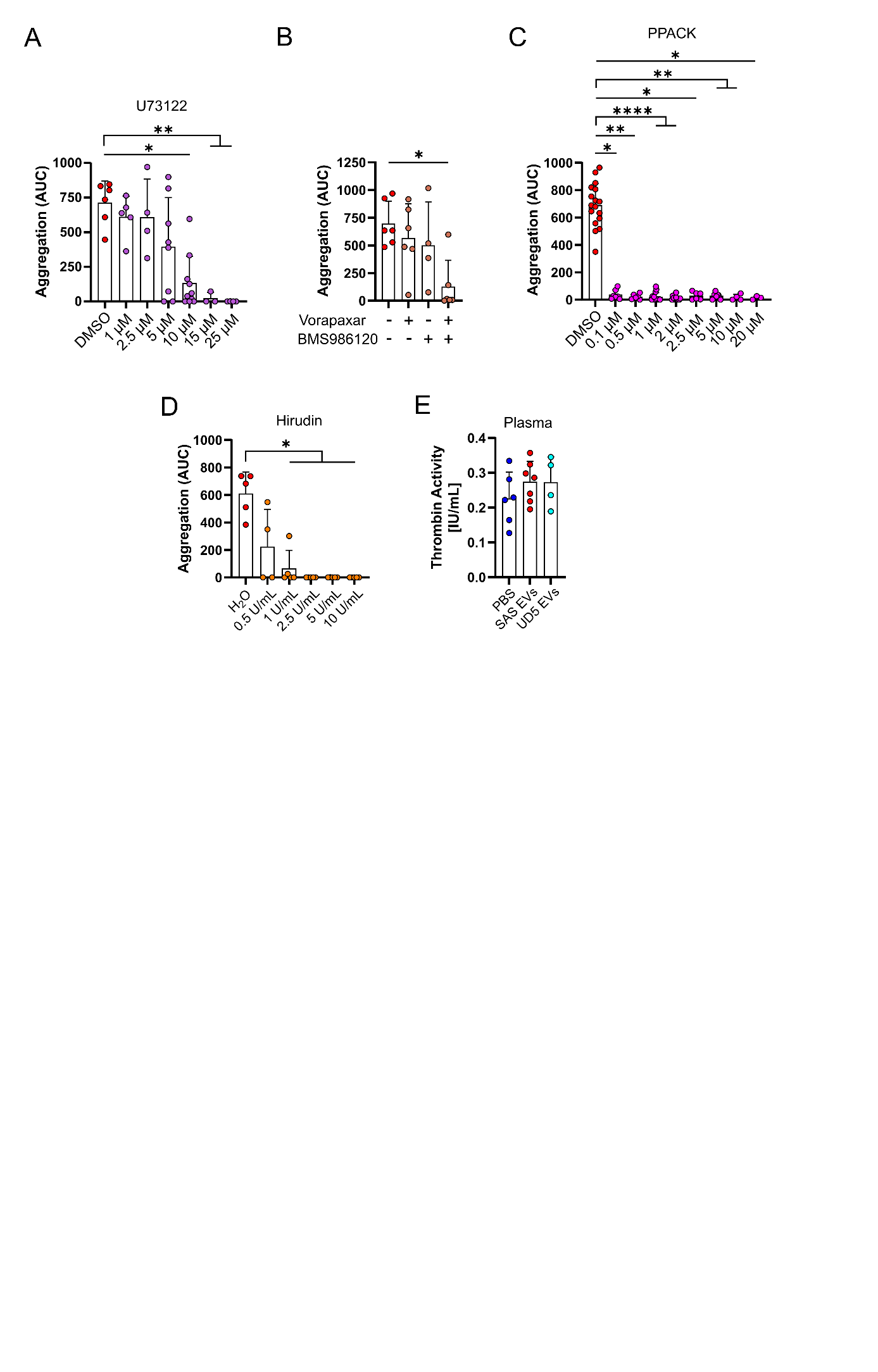
**

**Figure S2. Establishing effective inhibitors for EV-induced PLT aggregation.** PLTs and inhibitors were mixed in Tyrode’s buffer containing 2 mM Ca²⁺ within the aggregometer. After a 300-second pre-incubation, SAS-derived EVs (60 µg/mL) were added to induce aggregation and inhibitor efficacy was assessed. **(A)** A dose-response analysis of U73122, a PLC inhibitor, was performed to identify effective concentrations for suppressing EV-induced PLT aggregation. Multiple concentrations of U73122 (1–25 µM) were tested, with DMSO diluted 1:100 as the control (n = 3-10). **(B)** The effect of thrombin receptor inhibition on SAS EV-induced PLT aggregation was evaluated. Vorapaxar (PAR1 inhibitor, 10 µM) and BMS986120 (PAR4 inhibitor, 10 µM) were administered individually or in combination, with DMSO diluted 1:500 (-) as the control (n = 4-6). **(C)** The inhibitory effect of PPACK, a thrombin inhibitor, was assessed at concentrations ranging from 0.1–20 µM to determine its efficacy against EV-induced PLT aggregation. DMSO (1:20,000) was used as the control (n = 3-16). **(D)** Testing of different Hirudin (thrombin inhibitor) concentrations (0.5-10 U/mL) for inhibitory effect on SAS EV-induced aggregation of PLTs. H_2_O (1:100) served as control (n = 4-5). **(E)** The Thrombin activity (IU/mL) was measured in supernatants of human plasma after the addition of SAS- or UD5-derived EVs (60 µg/mL). Addition of PBS served as control (n = 4-7). Data are presented as mean + SD. Statistical significance is as follows: *p < 0.05, **p < 0.01, ****p < 0.0001. Kruskal-Wallis test followed by Dunn’s post-hoc test for (A-D).

**
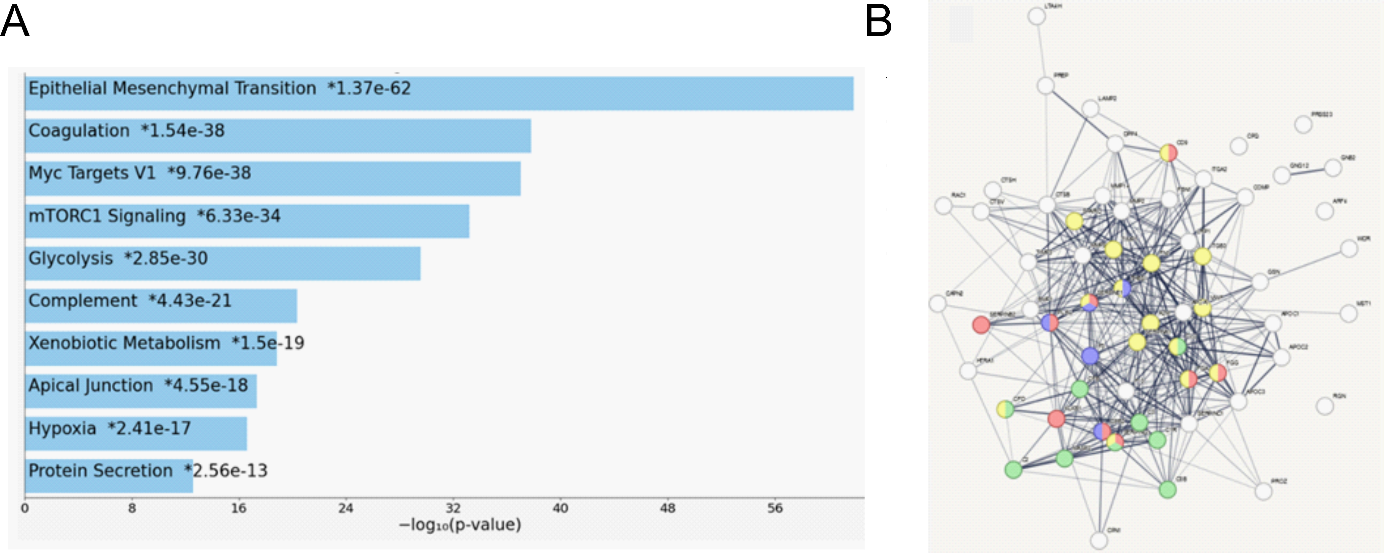
**

**Figure S3. Profiling of SAS EVs proteome.** **(A)** Bar chart showing the top enriched pathways, ranked by -log_10_(p-value), with the corresponding p-value displayed next to each term. An asterisk (*) next to a p-value indicates that the pathway also has a significant adjusted p-value (<0.05). The analyses were generated by Enrichr (https://maayanlab.cloud/Enrichr/) **(B)** Protein-protein interaction analysis of proteins involved in coagulation. Blue nodes represent proteins associated with positive regulation of coagulation, while red nodes represent those associated with negative regulation. Green nodes are complementing proteins, and yellow nodes represent PLT alpha granules. The analyses were generated by STRING database (https://string-db.org). The list of proteins is available in supplementary Table S3.


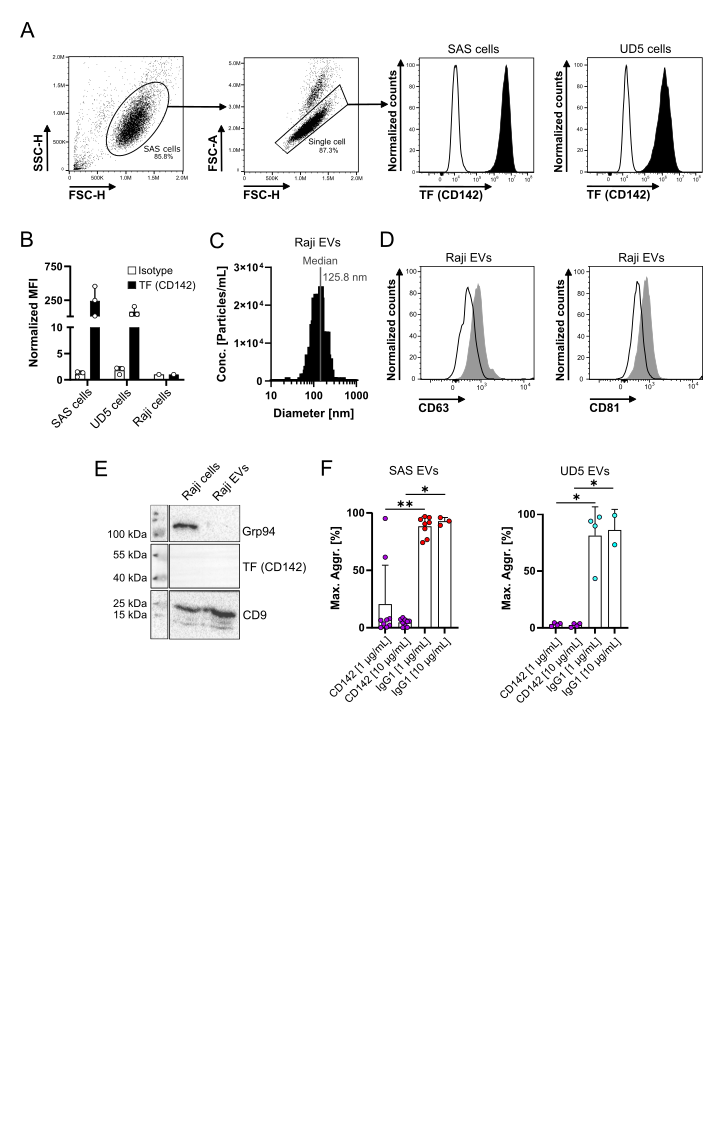


**Figure S4**. **Quantification of TF (CD142) expression on cell lines and EV characterization.** **(A)** Representative example of the gating strategy used to assess TF (CD142) expression on SAS and UD5 cells. The panels illustrate the gating strategy, showing cell populations in side scatter (SSC) and forward scatter (FSC) plots, followed by the selection of single cells. TF (CD142) expression is displayed in the histogram. Expression was determined by multiparameter flow cytometry, with black histograms representing TF (CD142) and white histograms (black line) representing isotype-matched controls. **(B)** Quantification of TF (CD142) expression on SAS, UD5, and Raji cells measured by flow cytometry. Mean fluorescence intensity (MFI) values were normalized to the average MFI of unstained samples. **(C)** Representative size distribution profile of Raji EVs, analyzed via NTA. **(D)** Representative flow cytometric histograms showing EV-specific surface markers on Raji EVs (gray histograms), labeled with FITC-conjugated CD63 and CD81 monoclonal antibodies. White histograms (black line) represent negative controls using isotype-matched monoclonal antibodies. **(E)** Western blot analysis of Raji cell lysates and Raji EV lysates. 20 µg of lysates were loaded per lane. The molecular weight markers (in kDa) provide size references, indicating the molecular weights of CD9 (~25 kDa), and GRP94 (~100 kDa). TF bands (~44 kDa) were not detected. **(F)** PLT aggregation response to EVs pretreated with anti-CD142 antibodies (concentrations indicated). Maximal aggregation is shown for SAS- and UD5-derived EVs (60 µg/mL; n = 3-9 SAS EVs; n = 2-4, UD5 EVs). All data are presented as mean + SD. Statistical significance is denoted as follows: *p < 0.05, **p < 0.01, determined using Kruskal-Wallis test followed by Dunn’s post-hoc test.
