# Supplementary material for: Head and neck squamous cell carcinoma-derived extracellular vesicles mediate Ca²⁺-dependent platelet activation and aggregation through tissue factor
Source: Cell Commun Signal. 2025 May 1;23:210. doi: 10.1186/s12964-025-02215-x (PMC12044835; doi:10.1186/s12964-025-02215-x)
Supplement: Supplementary file 4 — Supplementary Material 4 [file 12964_2025_2215_MOESM4_ESM.pdf]

Ponceau S staining (#5938.2, Roth; 0.05 g dissolved in 50 mL of 5% acetic acid in H_2O).
The ponceau blots serve as loading control for the uncut blots.

Ponceau S stainings related to the blots of figure 1E. I – CD9 blot, II – TSG101 blot.

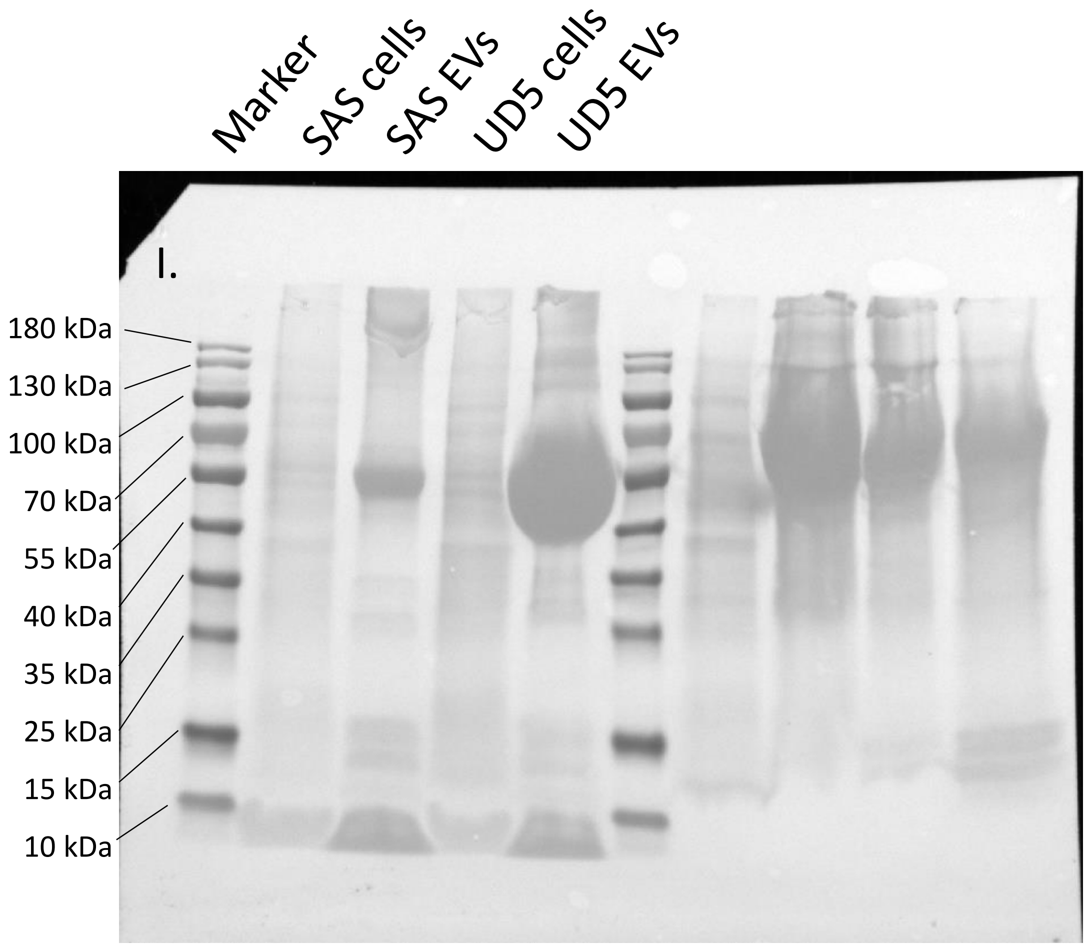

CD9 blot

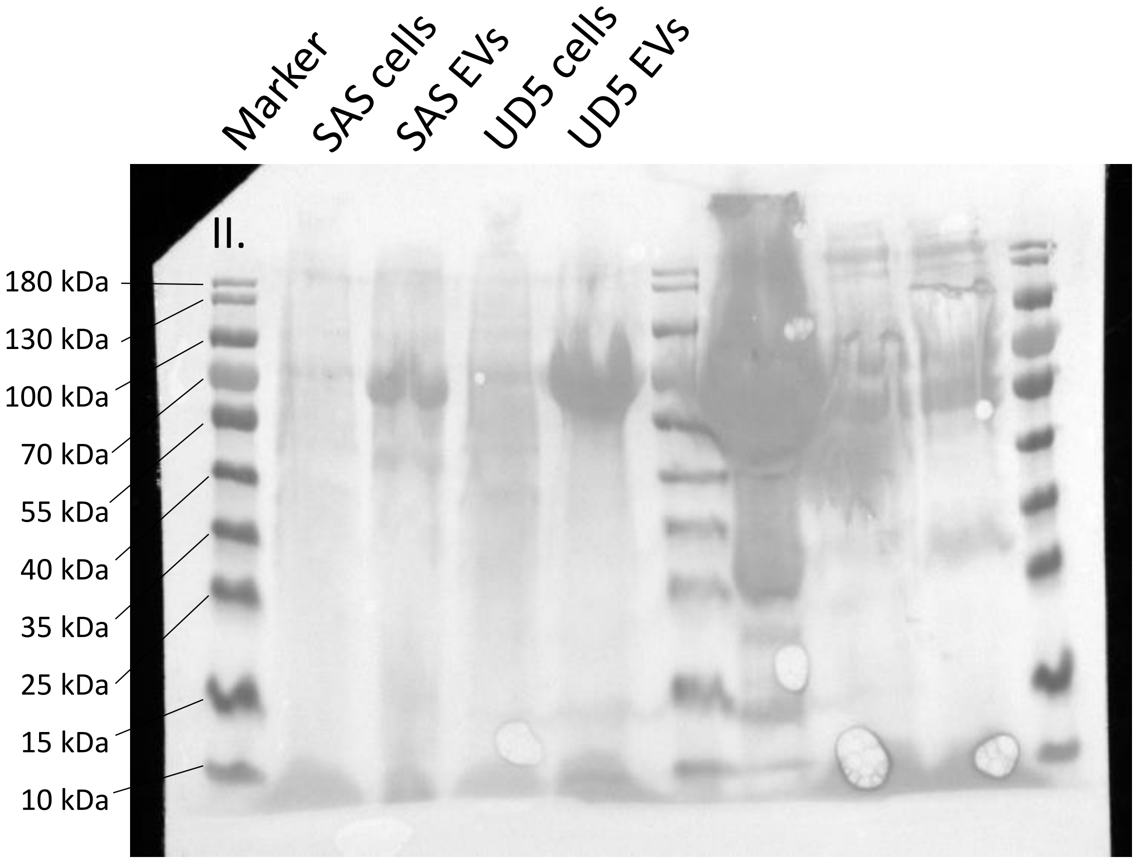

TSG101 blot

Ponceau S staining related to the blots of figure 1E. III – GRP94 blot

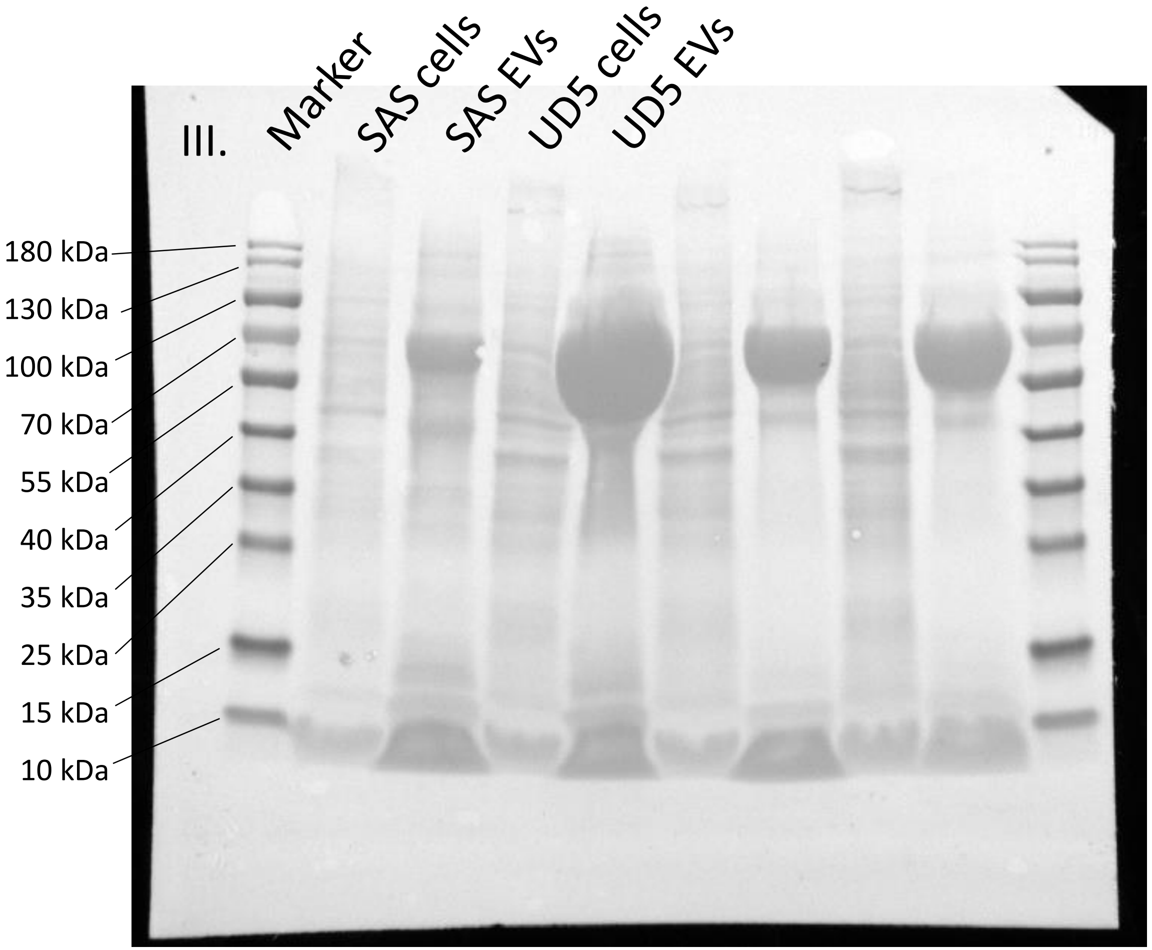

GRP94 blot

Uncut Western blots of figure 1E. The red boxes indicate the section, that was used in the manuscript. I – CD9, II – TSG101, III – GRP94

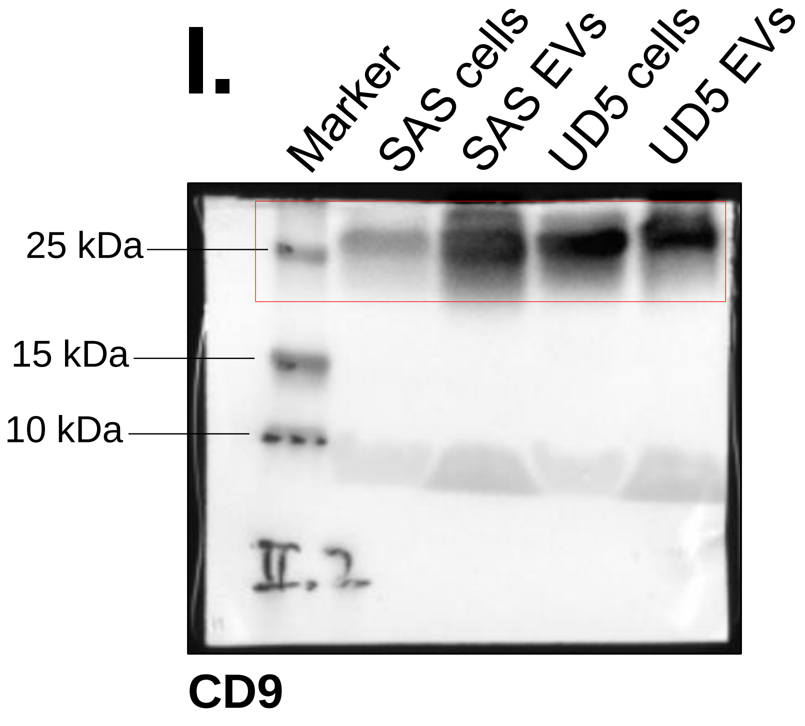

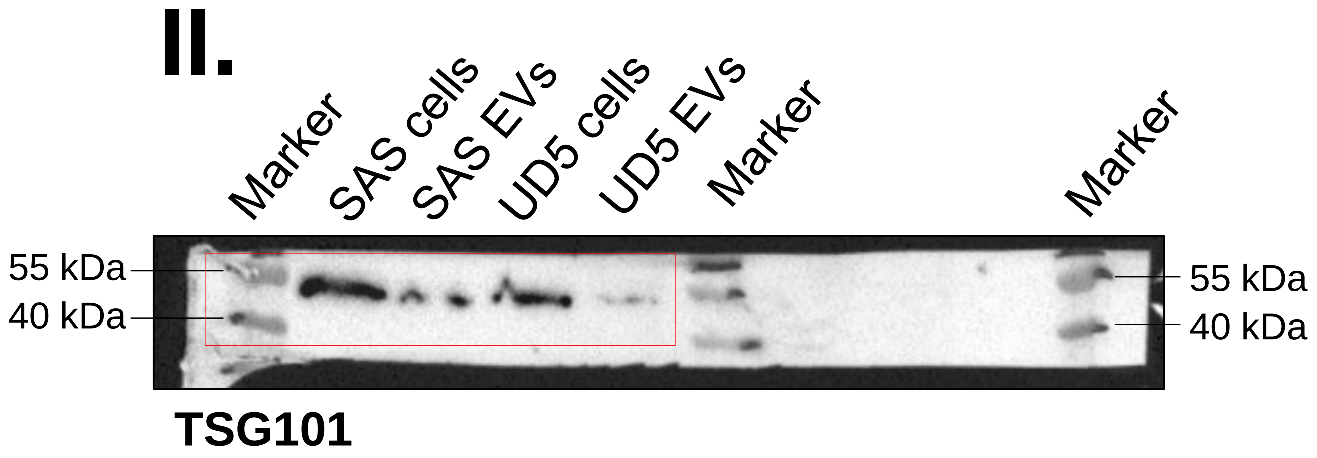

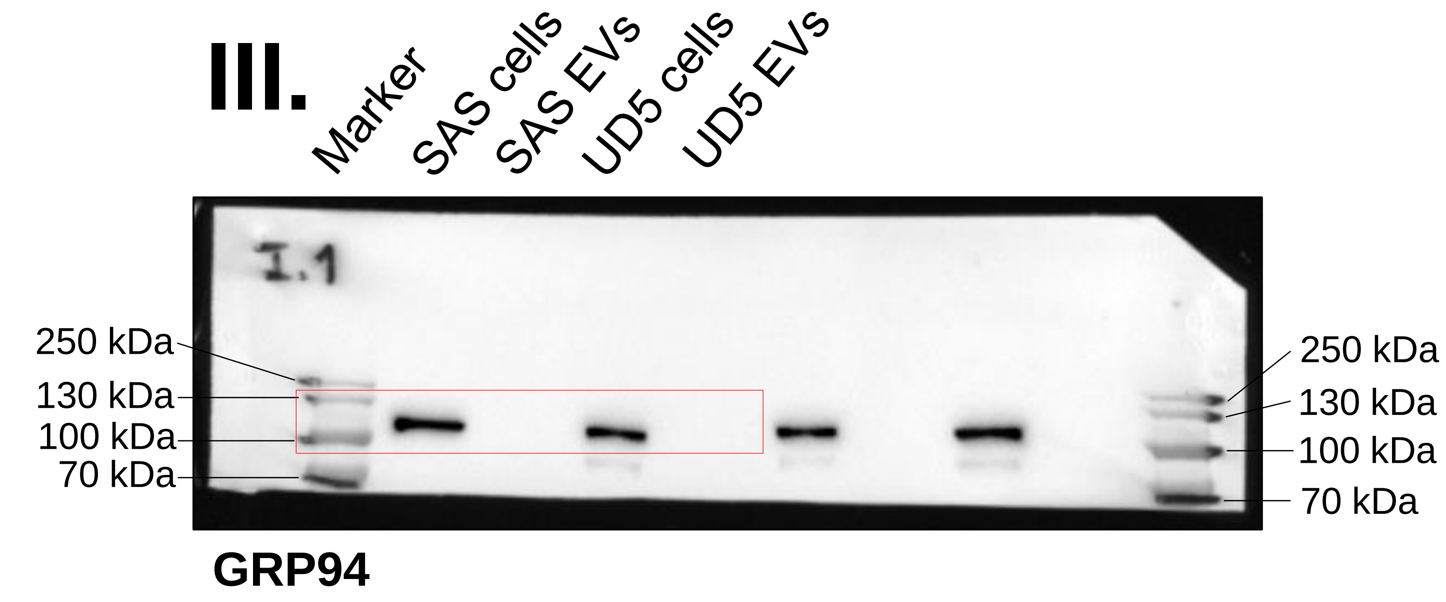

Ponceau S staining related to the blots of figure 5B.

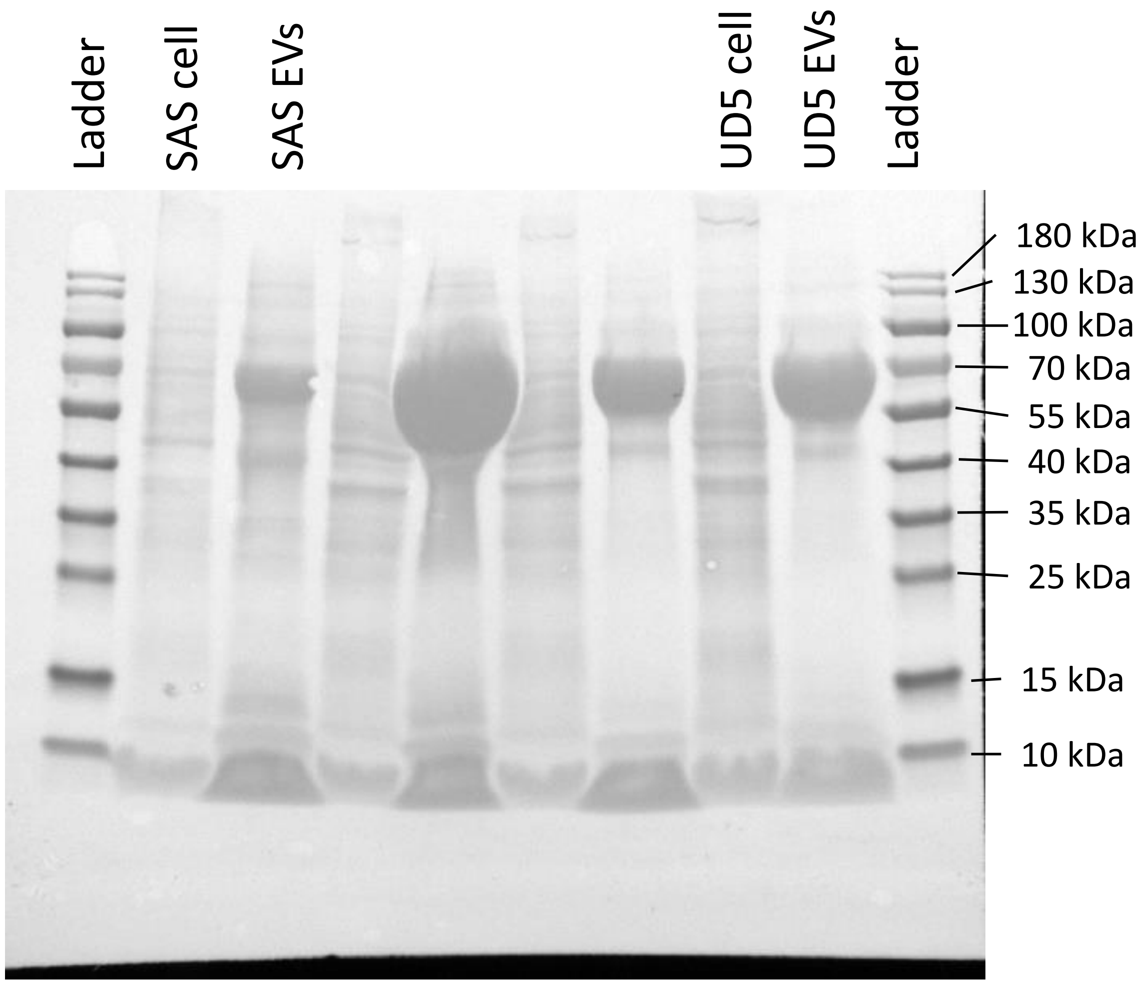

TF and GAPDH blot

Uncut Western blots of figure 5B. The red boxes indicate the section, that was used in the manuscript.

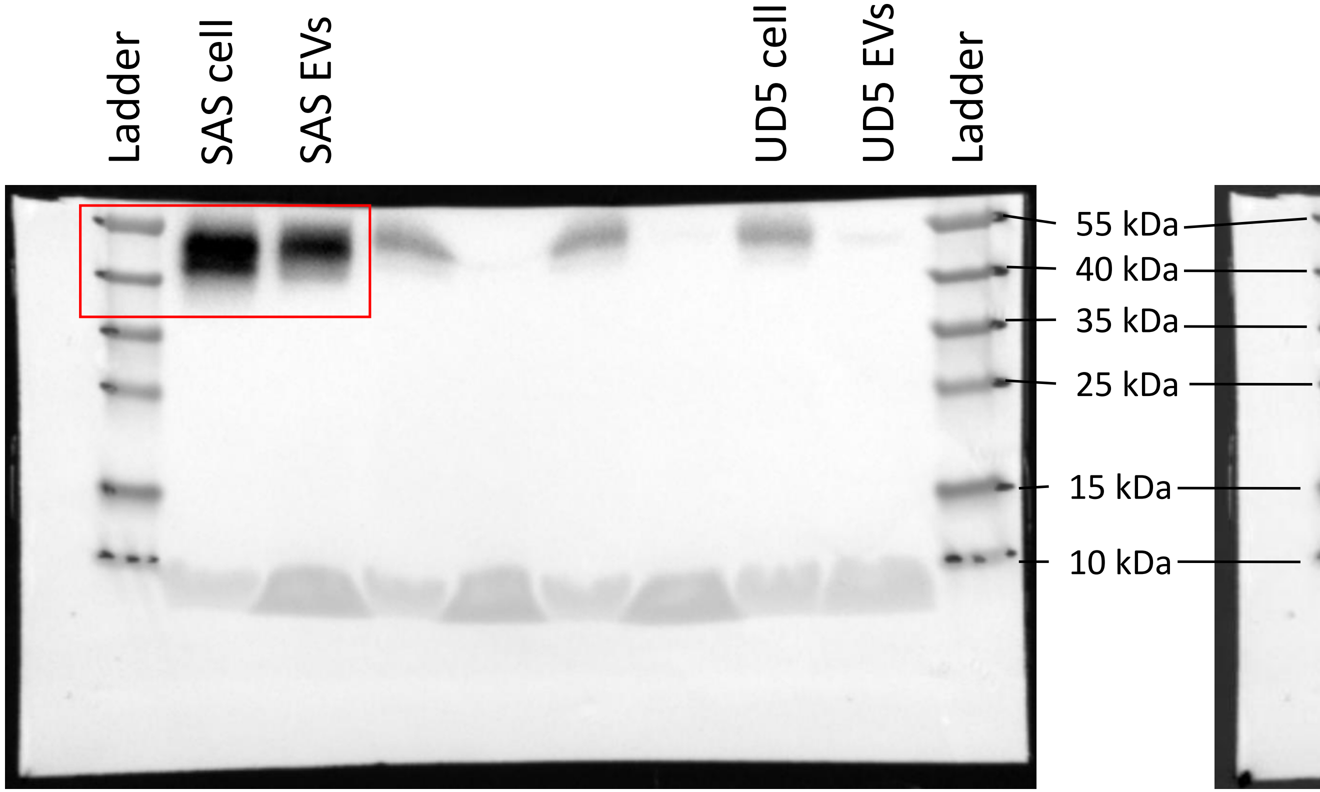

TF blot – 1 sec (SAS cells + EVs)

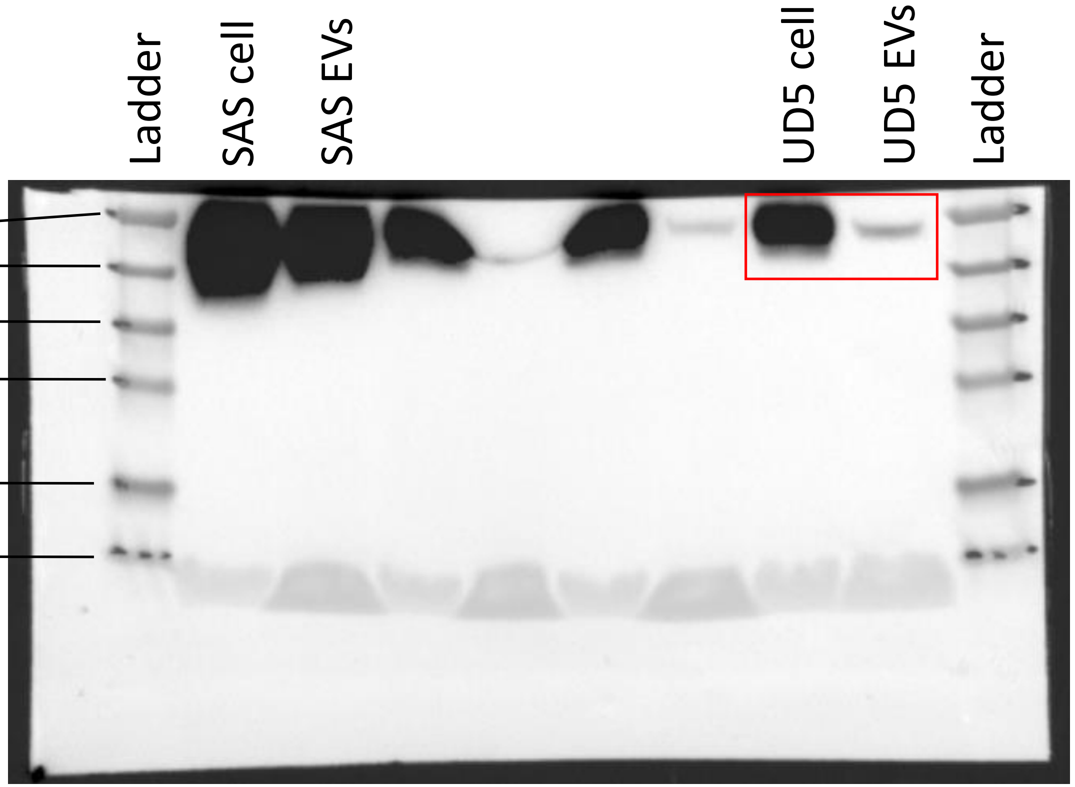

TF blot – 22.1 sec (UD5 cells + EVs)

Uncut Western blots of figure 5B. The red boxes indicate the section, that was used in the manuscript.

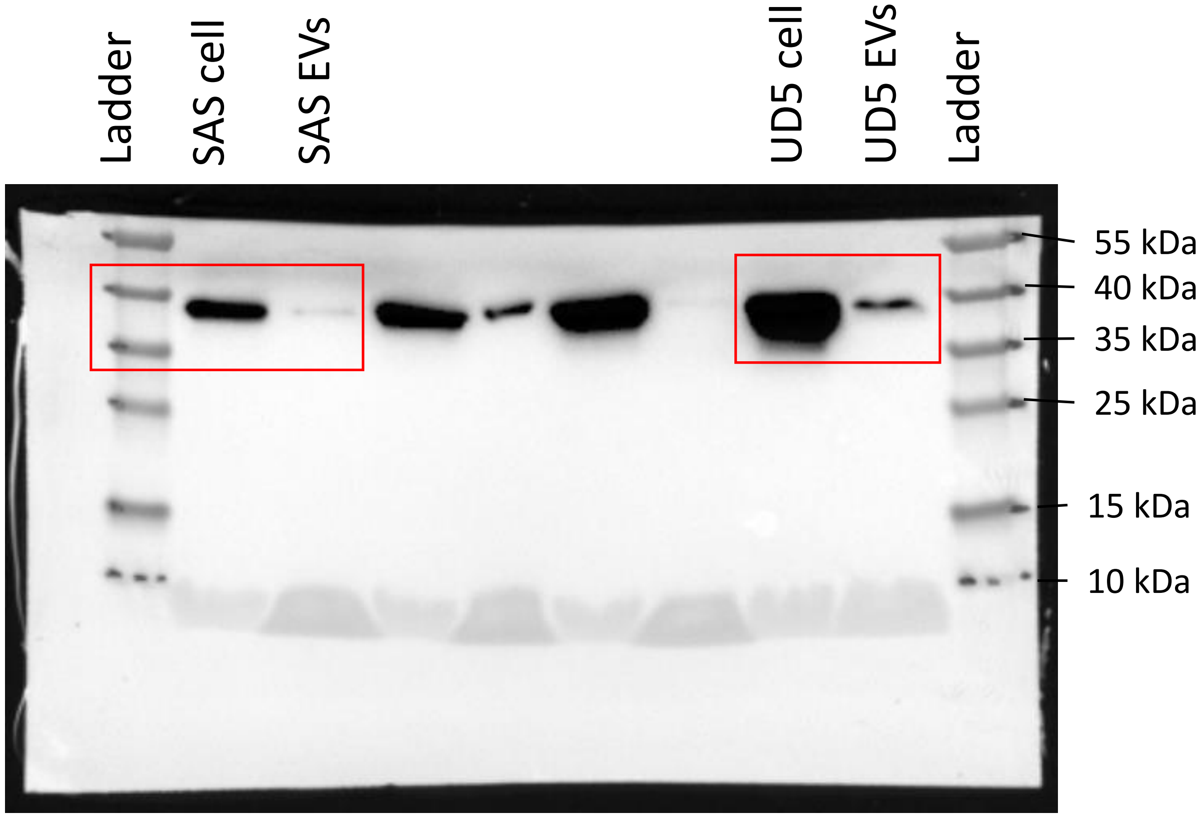

GAPDH blot

Ponceau stain related to the blot of figure S4E

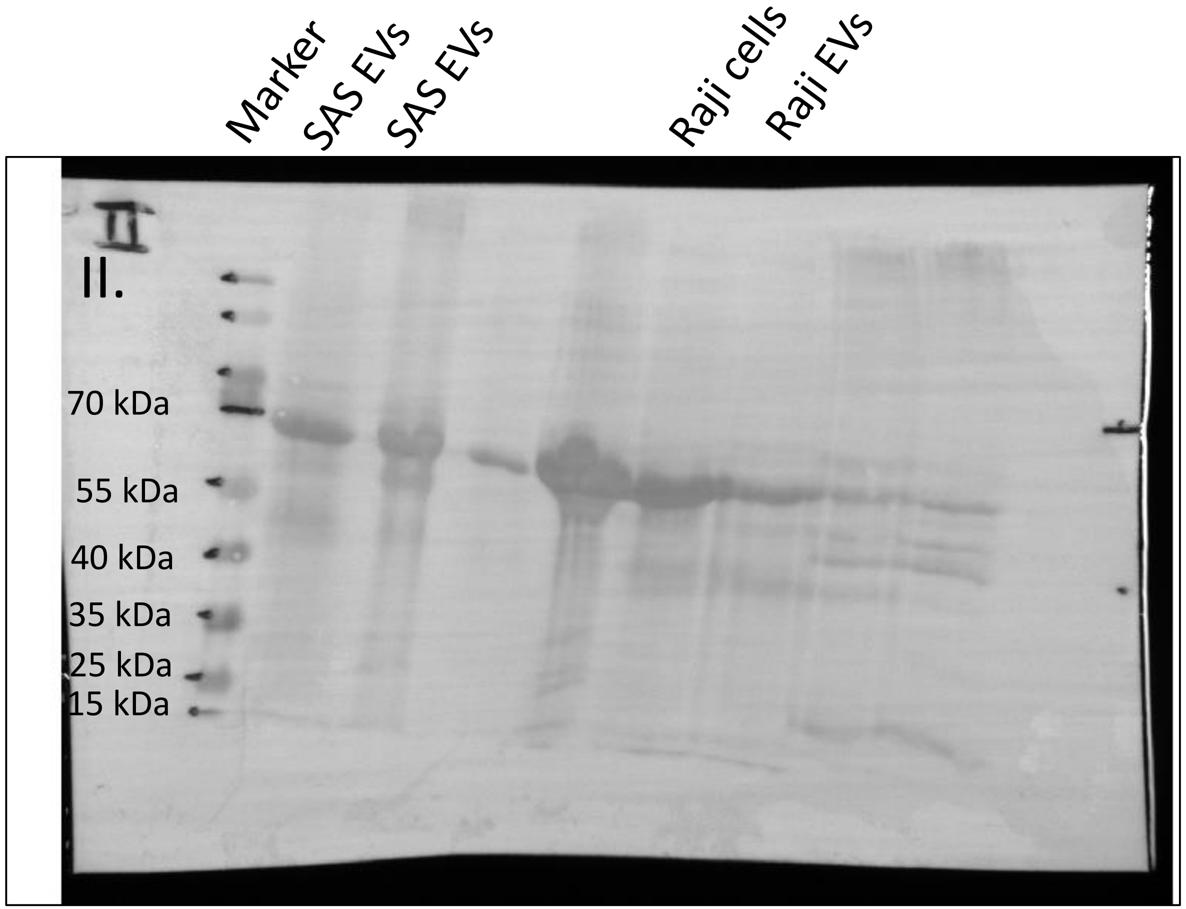

Ponceau stain

Uncut Western blots of figure S4E

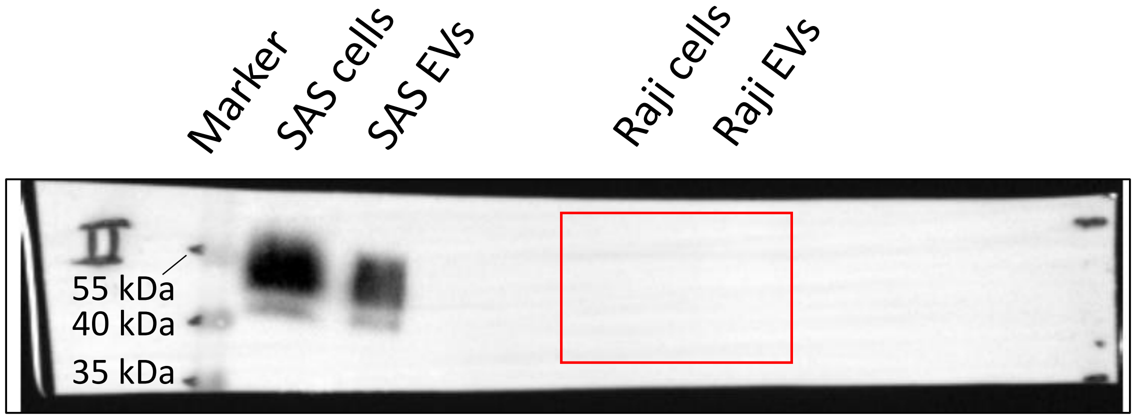

TF blot

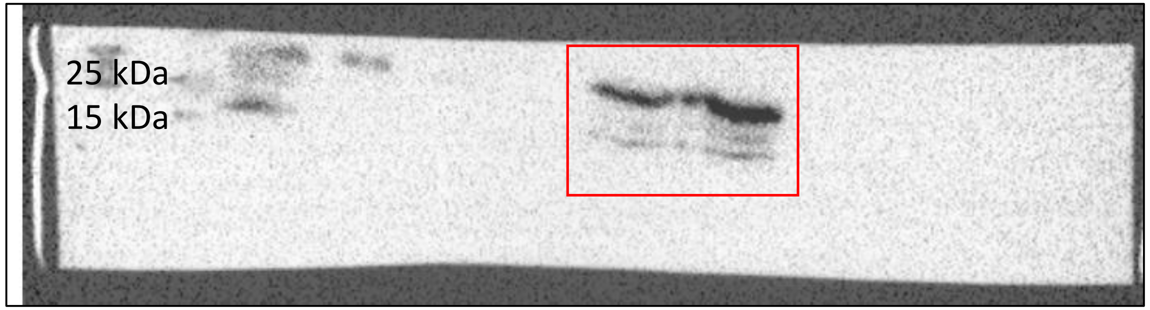

CD9 blot

Uncut Western blots of figure S4E. The red boxes indicate the section, that was used in the manuscript.

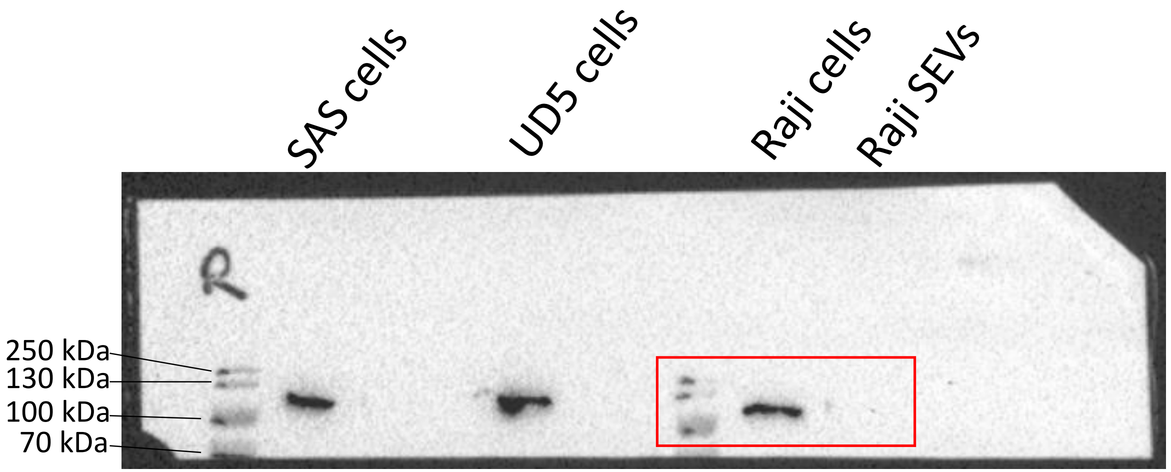

GRP94 blot
